# Toxicity-informed control of global PM_2.5_ emissions

**DOI:** 10.1093/nsr/nwag301

**Published:** 2026-05-21

**Authors:** Haotian Zheng, Shuxiao Wang, Xiangdong Li, Qing Li, Di Wu, Zbigniew Klimont, Dongxu Zhao, Lyuyin Huang, Zhaoxin Dong, Qingru Wu, Jingkun Jiang, Ling N Jin, Hong He, Kebin He, Jincai Zhao, Qian Liu, Scott Weichenthal, Aaron J Cohen, Jiming Hao

**Affiliations:** Nanjing-Helsinki Institute in Atmospheric and Earth System Sciences, Nanjing University, Nanjing 210023, China; State Key Laboratory of Regional Environment and Sustainability, School of Environment, Tsinghua University, Beijing 100084, China; State Key Laboratory of Climate Resilience for Coastal Cities, Department of Civil and Environmental Engineering, The Hong Kong Polytechnic University, Hong Kong, China; Frontiers Science Center for Critical Earth Material Cycling, Nanjing University, Nanjing 210023, China; State Key Laboratory of Regional Environment and Sustainability, School of Environment, Tsinghua University, Beijing 100084, China; Key Laboratory of Sources and Control of Air Pollution Complex, Ministry of Ecology and Environment, Beijing 100084, China; State Key Laboratory of Climate Resilience for Coastal Cities, Department of Civil and Environmental Engineering, The Hong Kong Polytechnic University, Hong Kong, China; Department of Environmental Science and Engineering, Shanghai Key Laboratory of Atmospheric Particle Pollution and Prevention, Fudan University, Shanghai 200433, China; Department of Environmental Science and Engineering, Shanghai Key Laboratory of Atmospheric Particle Pollution and Prevention, Fudan University, Shanghai 200433, China; Energy, Climate and Environmental Program, International Institute for Applied Systems Analysis (IIASA), Laxenburg 2361, Austria; Nanjing-Helsinki Institute in Atmospheric and Earth System Sciences, Nanjing University, Nanjing 210023, China; State Key Laboratory of Regional Environment and Sustainability, School of Environment, Tsinghua University, Beijing 100084, China; Key Laboratory of Sources and Control of Air Pollution Complex, Ministry of Ecology and Environment, Beijing 100084, China; State Key Laboratory of Regional Environment and Sustainability, School of Environment, Tsinghua University, Beijing 100084, China; School of Environment and Energy, South China University of Technology, Guangzhou Higher Education Mega Center, Guangzhou 510006, China; State Key Laboratory of Regional Environment and Sustainability, School of Environment, Tsinghua University, Beijing 100084, China; Key Laboratory of Sources and Control of Air Pollution Complex, Ministry of Ecology and Environment, Beijing 100084, China; State Key Laboratory of Regional Environment and Sustainability, School of Environment, Tsinghua University, Beijing 100084, China; Key Laboratory of Sources and Control of Air Pollution Complex, Ministry of Ecology and Environment, Beijing 100084, China; State Key Laboratory of Climate Resilience for Coastal Cities, Department of Civil and Environmental Engineering, The Hong Kong Polytechnic University, Hong Kong, China; Department of Health Technology and Informatics, The Hong Kong Polytechnic University, Hong Kong, China; Laboratory of Atmospheric Environment and Pollution Control, Research Center for Eco-Environmental Sciences, Chinese Academy of Sciences, Beijing 100085, China; State Key Laboratory of Regional Environment and Sustainability, School of Environment, Tsinghua University, Beijing 100084, China; Key Laboratory of Sources and Control of Air Pollution Complex, Ministry of Ecology and Environment, Beijing 100084, China; Key Laboratory of Photochemistry, Institute of Chemistry, Chinese Academy of Sciences, Beijing 100190, China; State Key Laboratory of Environmental Chemistry and Ecotoxicology, Research Center for Eco-Environmental Sciences, Chinese Academy of Sciences, Beijing 100085, China; Department of Epidemiology, Biostatistics, and Occupational Health, McGill University, Montreal H3A1G1, Canada; Health Effects Institute, Boston, MA 02108, USA; State Key Laboratory of Regional Environment and Sustainability, School of Environment, Tsinghua University, Beijing 100084, China; Key Laboratory of Sources and Control of Air Pollution Complex, Ministry of Ecology and Environment, Beijing 100084, China

**Keywords:** PM_2.5_ toxicity, global emission inventory, air pollution control, environmental inequity

## Abstract

Fine particulate matter (PM_2.5_) remains a leading environmental health risk, yet air pollution control policies typically assume equal toxicity across emission sources. Unravelling the unequal toxicities in global PM_2.5_ emissions can support more effective air pollution control. Here, we integrate cell-based toxicological profiles with global emission inventories to develop the first global dataset of toxicity-adjusted PM_2.5_ emissions. We show that global toxicity-adjusted emissions are dominated by residential solid-fuel combustion, and that hotspots of PM_2.5_ mass and toxicity diverge substantially, with the highest toxicities occurring largely in regions reliant on traditional biomass. Low-income countries exhibit disproportionately high toxicity-adjusted emissions relative to their energy use, revealing a strong global environmental inequity. Incorporating unequal toxicities reshapes emission-control priorities, shifting many countries from mass-dominated industrial or power sectors towards residential combustion. We propose a toxicity-informed framework for air pollution control, which is adaptable to diverse socioeconomic contexts and can enhance global health and sustainability.

## Teaser

Not all air pollutants are equally toxic. Our global toxicity-adjusted PM2.5 inventory reveals that household solid fuels drive the most harmful emissions, reshaping control priorities for cleaner and healthier air.

## INTRODUCTION

Ambient fine particulate (particulate matter with a diameter of 2.5 μm or less; PM_2.5_) causes substantial morbidity and premature mortality, threatening public health through various acute and chronic diseases [1,2]. To tackle this obstacle, over the recent decades, air pollution control policies worldwide have successfully delivered substantial reductions in total PM_2.5_ mass concentrations in an effort to protect public health [3,4]. However, PM_2.5_ remains a leading environmental risk factor [5]. In 2025, the World Health Organization (WHO) proposed a global target calling for a 50% reduction in air pollution-related health impacts by 2040 relative to 2015 levels [6]. Achieving such an ambitious goal will require localized control policies that strategically target region-specific emission sources contributing most to PM_2.5_-related health risks, which still remain unclear [7,8].

From a global and historical perspective, energy use and industrial activities have been central drivers of air pollution, yet their dominant sources and their chemical compositions differed markedly across regions and socioeconomic contexts. Examples include coal combustion-driven pollution events such as the smog in London, the UK, in 1952, vehicle exhaust-dominated photochemical smog in Los Angeles, the USA, in the 1970s, and complex haze pollution involving multiple emission sources in Beijing, China, in the 2010s [9–11]. Such regional variations in PM_2.5_ sources yield chemically distinct particles with substantial differences in toxicity and associated health risks [12–14]. Nevertheless, current air pollution control strategies assume equal toxicity per unit mass across all sources [15,16]. Put

another way, the current approach to regulating PM_2.5_ assumes that interventions that reduce total outdoor PM_2.5_ mass concentrations by 1 μg/m^3^ will have the same health benefits even if one reduces sea salt in PM_2.5_ and one reduces known human toxicants such as lead, arsenic, or polycyclic aromatic hydrocarbons (PAHs). While this assumption is demonstrably false (i.e. known human activity-generated carcinogens in PM_2.5_ are more harmful than natural sea salt), epidemiological studies have yet to provide comprehensive source-specific exposure–response functions to describe the health risks attributable to specific kinds of PM_2.5_ [17]. As a result, targeted control policies that could deliver the same health benefits for reduced costs (i.e. by targeting the most toxic forms of PM_2.5_) have not been implemented.

As a complementary line of evidence, cellular toxicological assays offer mechanistic and quantitative comparisons of source-specific toxicities, thereby informing health-oriented air quality management [18,19]. Using this approach, numerous studies have quantified the cellular toxicities of PM_2.5_ from major anthropogenic and biogenic sources and reached broadly consistent conclusions [20–22]. For example, aerosol toxicity is largely driven by toxic organics and metals, particularly from residential solid-fuel use, diesel engines, and metal-rich industrial sources such as iron and steel production [23–25]. Building on this, our previous work systematically quantified the toxicities of major anthropogenic PM_2.5_ sources and developed a toxicity-adjusted emission inventory for China [26], which enabled the identification of key contributors and regional disparities and provided insights into region- and source-specific air pollution control.

Given the pronounced global heterogeneity in energy and industrial structure and socioeconomic development, the dominant contributors to PM_2.5_ differed markedly across regions and countries [27,28]. In addition to disparities in source composition, variations in source-specific toxicities imply that toxicity-weighted PM_2.5_ emissions may exhibit even greater regional variations. Such variations are expected across high-income countries such as the USA, emerging economies like China and India, and low-income regions in sub-Saharan Africa. However, a global inventory of toxicity-adjusted emissions has not yet been established, leaving their major sources, spatial patterns, and driving factors largely unexplored. Addressing this important research gap would provide a toxicity-informed perspective on global air pollution, thereby supporting targeted and locally adapted control strategies worldwide.

Here, leveraging source-specific toxicological profiles and a global emission inventory, we established the first global inventory of toxicity-adjusted PM_2.5_ emissions in 2015. This dataset moves beyond conventional mass-based metrics for PM_2.5_ and enables a systematic evaluation of disparities in energy use, mass emissions, and associated toxicities across regions and socioeconomic contexts. By employing the toxicity-informed metrics at the global scale, our study provides a new framework for formulating more targeted and locally tailored air pollution control strategies to achieve cleaner and healthier air.

## RESUTLS

### Global inventory of toxicity-adjusted PM_2.5_ emissions

Building on our previous work in China [26], we constructed a global inventory of toxicity-adjusted PM_2.5_ emissions by integrating PM_2.5_ mass emissions with their source-specific toxicities. The toxicological profiles were derived from cellular assays using two widely applied biological endpoints with human epithelial A549 cell lines: oxidative stress (OS) and cytotoxicity (CT). We characterized source-specific toxicity using relative potencies (RPs), which are defined as the toxic potencies per unit PM_2.5_ mass compared with those of coal-fired power plants (CFPPS; elaborated in ‘Methods’ section), which exhibit the lowest toxic potencies among major sources tested. Relative potencies based on OS and CT endpoints are denoted as RP_OS_ and RP_CT_, respectively. In general, source-specific toxicities increase in the order of power plants (lowest), the cement industry, gasoline vehicles, diesel vehicles, brake wear, metallurgy industry, and residential combustion (highest).

**Figure 1.**
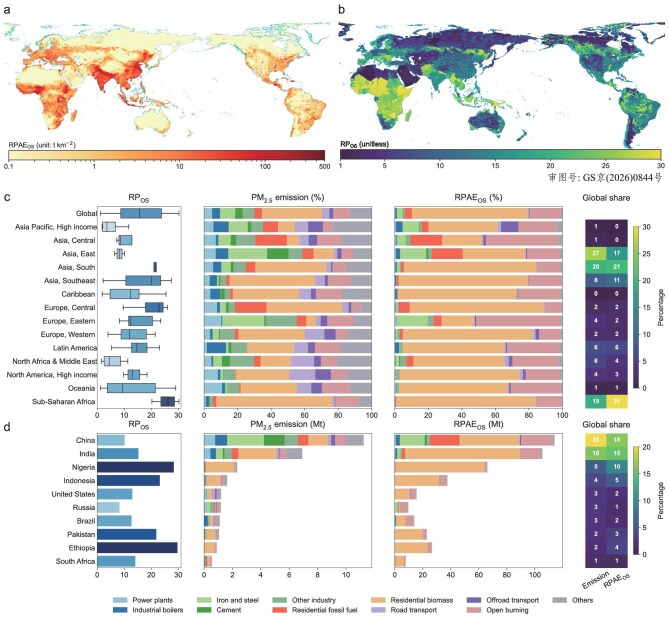
Global distributions and source contributions of toxicity-adjusted PM_2.5_ emissions in 2015. (a) Global distribution of RPAEs for OS (RPAE_OS_). (b) Global distribution of RP for OS (RP_OS_) of PM_2.5_ emissions. (c) Regional variation across 14 world regions and the global average. From left to right, the panels show region-specific RP_OS_, the sectoral composition of PM_2.5_ mass emissions, the sectoral composition of RPAE_OS_, and the global shares of regional contributions to PM_2.5_ emissions and RPAE_OS_. (d) National variation in source contributions for the top 10 countries ranked by PM_2.5_ mass emissions. From left to right, the panels show country-level sectoral PM_2.5_ mass emissions, sectoral RPAE_OS_, and the global shares of national contributions to total PM_2.5_ emissions and RPAE_OS_.

In 2015, the global relative potency-adjusted PM_2.5_ emissions (RPAE; elaborated in ‘Methods’ section) for OS and CT (RPAE_OS_ and RPAE_CT_, respectively) were 691.0 and 1107.0 million tons, respectively, with the RPs for OS and CT (RP_OS_ and RP_CT_, respectively) being 16.2 and 26.0, respectively. Figure 1a and b displays the spatial distributions of RPAE_OS_ and RP_OS_ of PM_2.5_ emissions worldwide. It is evident that the emission hotspots of RPAE exhibited similar distributions to PM_2.5_ mass (Fig. S1), whereas the hotspots of RPs of PM_2.5_ were decoupled from both PM_2.5_ mass and toxicity-adjusted emissions (i.e. the most toxic PM_2.5_ per unit mass are not necessarily where PM_2.5_ mass emissions are greatest). For example, mass emission hotspots like China and India demonstrated lower RPs than the global average. The comparisons between PM_2.5_ mass and RPAE_CT_ illustrate similar patterns (Fig. S2). These patterns are largely driven by differences in source compositions.

### Global and national source contributions to PM_2.5_ emissions and RPAE

Figure 1c shows RP_OS_ of PM_2.5_ emissions and the contributions of major sources to PM_2.5_ mass emissions and RPAE_OS_ at the global and regional scales (world regions defined in Table S1). Globally, the toxicities of PM_2.5_ emissions spanned over an order of magnitude, with RP_OS_ ranging from 1.2 to 30.2. Specifically, the high-income Asia Pacific region exhibited the lowest mass-weighted RP_OS_ (7.8), while sub-Saharan Africa demonstrated the highest (26.2). Several regions, including North and Latin America, Western Europe, South Asia, and Oceania, showed RP_OS_ values near the global average, with Oceania notable for the greatest intraregional variability. Such heterogeneity is mainly driven by differences in the sectoral composition of PM_2.5_ emissions across regions. With the markedly different intrinsic toxicities of PM_2.5_ emitted from major source categories, such as high-RP residential combustion, medium-RP industry, and low-RP power plants, regional contrasts in sectoral contributions translate into large differences in overall PM_2.5_ toxicity, as detailed below.

Globally, anthropogenic PM_2.5_ emissions were dominated by residential combustion of fossil fuel and biomass (40.3%), industry (25.2%), and open burning of biomass (10.2%). When weighted by RPs, residential combustion remained the predominant contributor, accounting for 73.5% of RPAE_OS_ and 80.0% of RPAE_CT_ (Fig. S2c). Industry and open burning also contributed substantially to anthropogenic PM_2.5_ emissions, though their shares were considerably lower than that of residential combustion and differed between the OS and cytotoxic potencies. In short, residential combustion, particularly biomass, dominated both PM_2.5_ mass and toxicity-adjusted emissions, yet its relative importance differed markedly among regions.

In general, regions with limited reliance on residential combustion showed lower toxicities, whereas those dominated by traditional solid fuels exhibited elevated toxicities. For example, residential combustion contributes only 11.5%–22.0% of emissions in Eastern Europe, high-income Asia Pacific, North Africa, and the Middle East, corresponding to RP_OS_ as low as 7.8–10.3. In contrast, residential combustion exceeded 50% in sub-Saharan Africa, Central Europe, and Southeast Asia, driving RP_OS_ as high as 17.7–26.2. These contrasts highlight the pivotal role of household fuel use in shaping global disparities in PM_2.5_ toxicity.

Nevertheless, South Asia and East Asia together accounted for 36.5% of global residential combustion emissions, yet their RPs were close to or below the global average. This apparent inconsistency arose from the substantial contributions of lower-toxicity sources such as power plants, cement production, and road transport, which moderated the overall toxicity of emissions in these regions. Therefore, East Asia emitted 26.6% of global PM_2.5_ mass but contributed only 16.6% to RPAE_OS_, whereas sub-Saharan Africa emitted 19.4% of mass but accounted for 31.5% of RPAE_OS_. The statistical relationships between sectoral contributions and RPs further confirm that residential combustion is strongly and positively associated with toxicity (Fig. S3).

At the national level, China and India dominated global PM_2.5_ mass emissions, contributing 26.4% and 16.2%, respectively, and also ranked first and second in RPAE_OS_ and RPAE_CT_ (Fig. 1d and Fig. S2d). However, both countries exhibited RPs below the global average, coinciding with a larger share of lower-toxicity sources such as power generation and industry. For example, power plants and industry accounted for 58.8% of PM_2.5_ mass emissions in China, leading to lower RP_OS_. In contrast, Nigeria and Ethiopia recorded extremely high RP_OS_ (28.3 and 29.7), driven by their overwhelming reliance on residential biomass (86.2% and 83.4%, respectively). Russia presented the opposite case: although it ranked sixth in PM_2.5_ mass emissions, its low dependence on residential combustion (8.8%) and high source contribution from power plants and industry (59.0%) resulted in the lowest RP among the top ten countries, placing it only 12th in RPAE_OS_ (Supplementary Data).

### Global disparities in PM_2.5_ emissions and toxicities

**Figure 2.**
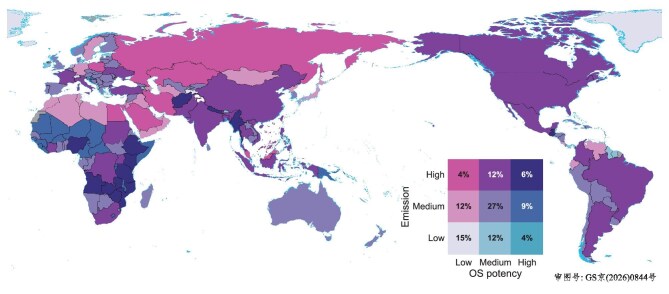
Global distribution of national PM_2.5_ emissions and RP_OS_. Countries were grouped into low, medium, and high categories using thresholds of 15 and 150 kilotons for PM_2.5_ emissions, and 10 and 25 kilotons for OS potency (RP_OS_). The colour scale shows each country’s combined category, and the percentages in the legend indicate the proportion of countries in each category.

Extending this analysis to all countries, we further examine how PM_2.5_ emission magnitude and intrinsic toxicity jointly shape systematic global disparities. The global map of PM_2.5_ emissions and RP_OS_ (Fig. 2) reveals a pronounced spatial discrepancy between PM_2.5_ mass emissions and toxicities. High mass-emission countries were distributed across East and South Asia, North America, and sub-Saharan Africa, with additional clusters in Europe and Latin America. Their large mass emissions were mainly driven by population size, fuel consumption, and industrial activity. In contrast, PM_2.5_ toxicity hotspots were predominantly concentrated in sub-Saharan Africa and in a few South Asian countries.

Among high-emission countries, several showed low toxicities, such as Russia, Egypt, Poland, and Iran. The low toxicities were attributable to the fact of low dependence on household solid fuels, whereas the high emissions were often driven by population size, with per-capita emissions usually remaining relatively modest (country- and region-level source-specific PM_2.5_ emissions are provided in the Supplementary Data). For example, Egypt fell into the low-emission/low-toxicity category when examined from a per-capita perspective (Fig. S4). In contrast, at the other extreme, a few countries demonstrated the opposite pattern: low PM_2.5_ emissions but markedly high toxicities (e.g. The Gambia). While their high toxicities largely stemmed from dependence on residential solid fuels, their low emissions were attributable to the small population sizes. As a result, most countries no longer belonged to this category when viewed on a per-capita basis.

A critical cluster of countries with concurrent high emissions and high toxicity was concentrated in sub-Saharan Africa (e.g. Nigeria, Ethiopia, Kenya, and Tanzania) and in parts of South and Southeast Asia (e.g. Nepal, Myanmar, and Afghanistan). This was attributable to large population sizes, less developed industrial bases, and heavy dependence on traditional biomass fuels. In these regions, people are subject to both high levels of PM_2.5_ exposure and elevated toxicity. Importantly, populations in many of these countries are also rapidly growing, which may lead to continued increases in emissions with high toxicities in the coming decades. Meanwhile, these populations largely lacked access to clean energy and faced affordability constraints, with residential biomass remaining the dominant fuel, suggesting that the ‘double-burden’ (i.e. high emissions and high toxicity) situation will persist unless substantial energy transitions occur.

Many sub-Saharan states fell into the medium-emission but high-toxicity category. Their energy structures resembled those of ‘double-burden’ countries, leading to elevated toxicities. In contrast, a group of high-emission countries, including major economies such as China, the USA, and India, as well as several emerging economies like Brazil, Indonesia, and Vietnam, exhibited moderate toxicities. In these countries, despite their considerable energy consumption, more diversified energy and technology structures largely offset the impact of household solid fuel use, thereby lowering the overall toxicity of emissions. Together, this group accounted for 74.0% of global PM_2.5_ emissions and 64.1% of RPAE_OS_.

Among the medium-emission/low-toxicity countries, two distinct groups were observed. The first group consisted of several Middle Eastern and North African countries, such as Qatar, the United Arab Emirates, and Libya, where energy systems were dominated by oil and natural gas. In these countries, the near absence of household solid fuel use kept toxicities low, even though overall emissions remained at medium levels. The second group comprised industrialized European countries, including Germany, the Netherlands, and Sweden. In these countries, abundant clean energy supplies and stringent industrial emission controls constrained the toxic potencies of PM_2.5_ emissions.

A further group of small countries, such as Switzerland and Iceland, exhibited both low emissions and low toxicities. Beyond clean energy use and favourable industrial structures, their heavy reliance on imported goods suggests that some high-toxicity emissions were displaced to trading partners via international trade [29], which indicates that the double-low conditions are highly context-specific and not easily generalizable globally.

### Socioeconomic gradients in energy use, PM_2.5_ emissions, and toxicities

**Figure 3.**
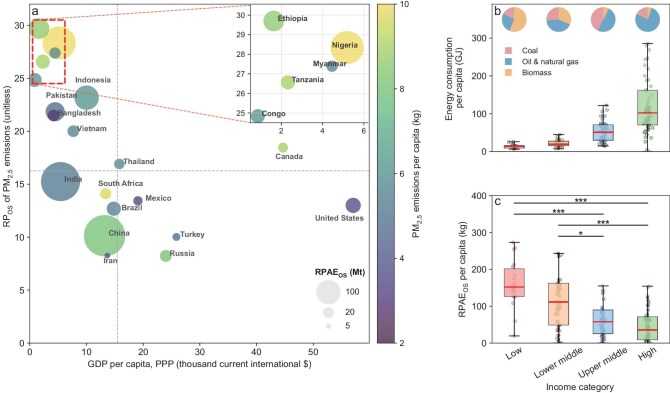
Global disparities in energy use, PM_2.5_ emissions, and RPAEs for OS (RPAE_OS_) across income groups in 2015. (a) Relationship between GDP per capita [as PPP, current international dollars, obtained from the World Bank (https://data.worldbank.org/)] and RP for OS (RP_OS_) of PM_2.5_ emissions for the top 20 emitting countries, with bubble size proportional to RPAE_OS_ and colour indicating per-capita emissions. The dashed lines indicate the global average GDP per capita (15 470 dollars) and RP_OS_ (16.2). The inset highlights countries with exceptionally high toxicities. (b) Boxplot of energy consumption per capita (GJ) across World Bank income categories. Data are plotted as box-and-whiskers (the line of the box is the median, the edges of the boxes are the 25th and 75th percentiles, and the lengths of the whiskers are within 1.5 times the interquartile range). Each box plot represents the distribution within the income category. Pie charts show the relative shares of coal, oil and natural gas, and biomass by income category. (c) Boxplot of RPAE_OS_ per capita (kg) by income category.

Beyond geographic disparities, we examined socioeconomic patterns by analyzing the relationship between national income levels and PM_2.5_ emissions and toxicities. Among the top 20 countries in PM_2.5_ emissions (Fig. 3a), RP_OS_ showed a significant negative correlation with gross domestic product (GDP) per capita (*P* < 0.05). Low-income countries, such as Ethiopia, exhibited the highest RP, while high-income economies, such as the USA, displayed substantially lower RP. Several middle-income countries, such as India and Vietnam, fell in between with medium RP. This negative income–toxicity relationship was observed consistently across all world regions (Fig. S5), e.g. Western and Eastern Europe, North Africa, and the Middle East exhibit higher income levels but lower RPs than the global average. In contrast, national and regional PM_2.5_ emissions and RPAE_OS_ did not vary systematically with income: the major contributors spanned all income levels, largely affected by the population size and energy structure.

To further clarify the relationship between income and PM_2.5_ emissions and RPAE, we examined the per-capita energy use and emissions across World Bank income categories (Table S2). We found that per-capita energy use increased about 10-fold from 15.6 GJ in low-income countries to 148.6 GJ in high-income countries, whereas per-capita RPAE decreased significantly from about 159.1 to 49.6 kg (*P* < 0.001; Fig. 3b and c). Such a striking inversion indicates a decoupling between energy consumption and RPAE: people in high-income countries consumed substantially more energy yet incurred markedly lower toxicity-adjusted emissions. To disentangle these disparities, we decomposed per-capita RPAE into three key drivers: per-capita energy use, emission intensity (PM_2.5_ emissions per unit of energy use), and RP of emissions. This analysis is based on cross-sectional country-level data for 2015 and therefore characterizes global disparities at this time point rather than their temporal evolution.

In terms of per-capita energy use, beyond the increasing trend across income groups, we also observed a salient difference in energy structure. Low-income groups relied heavily on biomass (56.3% of total energy), with smaller shares of oil (26.3%) and coal (17.4%). From low- to high-income countries, biomass shares declined steeply, reaching only 5.3% in high-income countries, while oil and natural gas together accounted for 77.2%. These structural disparities became even more pronounced in the residential sector. Per-capita household energy use was broadly similar in low- and middle-income countries (LMICs) (8–9 GJ), but rose sharply in the high-income group to 22.0 GJ (Fig. S6). In addition, low-income countries relied overwhelmingly on biomass (89.9%; 6.7% coal, 3.4% oil), whereas high-income countries depended predominantly on oil and natural gas (86.1%). Such substantially distinct energy structures partially accounted for the variability in emission intensity and RP of emissions.

As for emission intensity, we found substantially higher values in low-income countries than in high-income countries, a disparity driven by two main factors (Fig. S7a–c). First, in low-income countries, residential biomass combustion dominated energy use and, lacking emission controls, produced much higher emissions per unit of energy than oil and natural gas. Second, in high-income countries, in contrast, although there were more energy- and pollution-intensive industries, advanced end-of-pipe control measures effectively reduced their emission intensities. As a result, the emission intensity in low-income countries and lower-middle-income countries was 14.7 and 9.2 times that in high-income countries. Therefore, despite stark contrasts in energy consumption, per-capita PM_2.5_ mass emissions remained broadly comparable across income groups with no significant differences (Fig. S7d).

Regarding RP of PM_2.5_ emissions, we found substantial differences across income groups (Fig. S8a and b), which were consistent with the patterns shown in Fig. 3a. Low-income countries exhibited the highest RP, primarily due to biomass-dominated household combustion that substantially elevated toxicity and weak industries. Middle-income countries faced a dual burden: they relied heavily on solid household fuels while also hosting heavy-polluting industries such as iron and steel production, which together contributed to RP levels well above global averages. In contrast, high-income countries showed the lowest RP, owing to their reliance on clean household fuels and the adoption of advanced industrial technologies combined with stringent emission controls. Consequently, countries with high residential energy shares, often LMICs with limited overall energy use, tended to have emissions characterized by markedly higher RP levels (Fig. S9).

Accounting for differences in RP fundamentally reshapes the cross-income patterns of emission burdens. The per-capita energy use in low-income countries amounted to only about one-tenth of that in high-income countries, yet their RPAE per unit of energy was more than 30 times higher for both OS and cytotoxic potencies. This stark contrast translates into a more than 3-fold gap in per-capita RPAE between the two groups, revealing a strong inversion between energy consumption and toxicity-adjusted emissions (Fig. 3c and Fig. S8c).

We further assessed the inequities in energy use, emissions, and RPAE worldwide using concentration curves and concentration index (CI; see ‘Methods’ section; Fig. S10). The CI value of energy consumption was 0.42, which was close to that of income (0.48), indicating that energy use was highly skewed towards wealthier countries. In contrast, the CI value of anthropogenic PM_2.5_ emissions was slightly negative (−0.06), reflecting relatively small inequity and a tendency for low-income countries to emit more than high-income countries. The inequity became considerably stronger when emissions were weighted by toxicity: the CI values of RPAE_OS_ and RPAE_CT_ were −0.23 and −0.24, respectively, showing a pronounced inversion relative to energy use. At the distributional extremes, the lowest-income 20% of the global population consumed only 5.5% of total energy but accounted for roughly 40% of toxicity-adjusted emissions, whereas the highest-income 20% consumed nearly half of global energy yet contributed only about 10% of RPAE. Overall, these findings reveal a profound energy and environmental inequity, with those consuming the least energy bearing the greatest toxicity-adjusted emissions.

### Toxicity-informed policy implications

**Figure 4.**
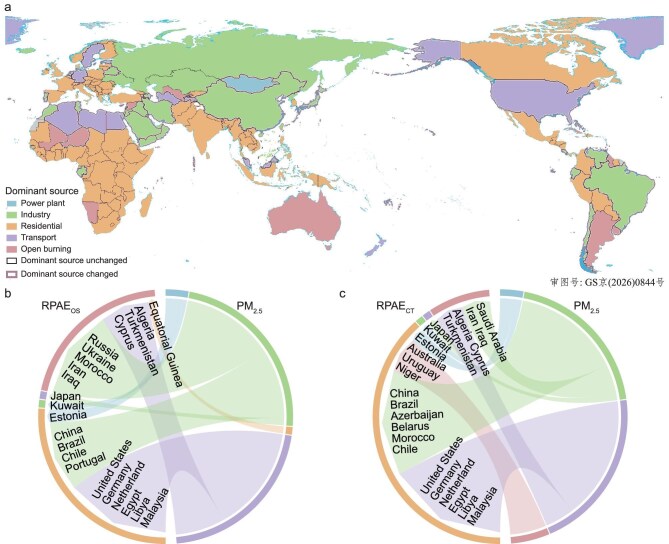
Geographic distribution and shifts in dominant sources of PM_2.5_ emissions. (a) Country-level dominant source category of national PM_2.5_ mass emissions in 2015. Countries whose dominant source category changes in both RPAEs for OS and CT (RPAE_OS_ and RPAE_CT_) are outlined with highlighted borders on the map. Shifts in dominant sources between PM_2.5_ mass and RPAE: (b) RPAE_OS_; (c) RPAE_CT_. The chord diagrams display only countries where the dominant source changed, while countries with unchanged dominant sources are not shown. The right side indicates the PM_2.5_ mass-based dominant source, while the left side shows the RPAE-based dominant source. Arc lengths do not represent emission magnitude; each country is shown with equal weight to indicate the direction of dominant-source shifts. Country names are labelled for selected examples rather than for all countries in each category.

The global toxicity-adjusted inventory of PM_2.5_ emissions enables a comparative assessment of sources and regions based on toxicity-weighted metrics. Our findings reveal a discrepancy between PM_2.5_ mass and toxicity: sources with the largest emissions are not necessarily those with the highest RPAE, indicating that mass-based emission controls are inefficient in terms of reducing population exposures from sources with potentially higher health risks. Extending conventional mass-based policies to toxicity-weighted metrics reveals critical shifts in national and regional priority sources (Fig. 4), highlighting the need to recalibrate control strategies to achieve greater health gains.

In low- and lower-middle-income countries, limited accessibility, affordability, and reliability of clean fuels kept household demand anchored in traditional solid fuels, particularly biomass [30,31]. This resulted in high PM_2.5_ mass emissions and even higher RPAE despite low overall energy use, trapping these disadvantaged populations in high-toxicity energy use devoted to basic subsistence. In this situation, early substitutions in the residential sector, e.g. replacing traditional biomass with electricity, liquefied petroleum gas, or natural gas, can yield substantial RPAE reductions and considerable energy and environmental justice gains with minimal changes in total energy consumption [32,33]. However, without adequate subsidies and infrastructure, such transitions may exacerbate inequities in energy burden, as poorer households face disproportionate costs for essential energy services [34]. Addressing this imbalance requires coordinated policies and international assistance to ensure that the health benefits of cleaner fuels do not come at the expense of economic hardship. Thus, for most low- and lower-middle-income countries, such as India, Vietnam, Nigeria, and Pakistan, residential biomass control remains a dual priority under both mass- and toxicity-based strategies, demanding sustained domestic and international investment.

In many upper-middle-income countries, PM_2.5_ mass emissions were dominated by industrial or power sectors, yet, once toxicity was considered, the leading sources shifted towards residential combustion (Fig. S11). Such transitions were evident in China and Brazil, where residential combustion surpassed industry in toxicity-weighted metrics (Figs 1d and 4b and c). A similar pattern was observed in high-income countries: 28 out of 67 countries were dominated by residential combustion for PM_2.5_ mass emissions (e.g. the UK, France, and Canada). However, when toxicity was taken into account, many countries that were previously dominated by industrial or transport sources for mass emissions (e.g. the USA and Germany) became residential-dominated. These upper-middle- and high-income countries generally possess stronger governance capacity, more developed infrastructure, and greater fiscal flexibility, such that clean energy transitions in household fuels are often technically feasible and can be pursued in socially equitable ways. Thus, focusing policy efforts on residential combustion could yield substantial health benefits and complement existing controls on energy and industry.

Beyond residential emissions, the dominance of energy and industrial sectors also exhibited substantial realignment when toxicity was taken into account. Under the mass-based framework, PM_2.5_ mass emissions of 34 countries were dominated by energy or industry, but this number declined to only 12 after incorporating cytotoxic weighting. Most of these countries fell within the upper-middle- and high-income groups, including Russia, Ukraine, the United Arab Emirates, and Qatar, where heavy industry or fossil-fuel-based power generation remained central to their economies. The consistency between mass- and toxicity-based dominance indicates that emissions from these sectors were substantial both in terms of quantity and intrinsic toxicity in these countries. Further reducing their impacts of toxicity by enhancing end-of-pipe control measures and upgrading energy and technological structures—such as transitioning to renewable-based power generation and adopting cleaner industrial processes (e.g. replacing basic oxygen furnaces with electric arc furnaces in steelmaking)—would jointly advance cleaner and healthier air.

The transport sector also exhibited a marked reduction in dominance when toxicity was considered. Among 31 countries that were transport-dominated for PM_2.5_ mass emissions, only 10 remained so after incorporating cytotoxic weighting. All of these were high-income economies, mostly island or coastal nations such as Singapore and Greenland. Notably, Japan’s dominant source shifted from industry to transport when toxicity was considered. Among transport sources, diesel vehicles contributed a substantially larger share of PM_2.5_ mass than gasoline vehicles and exhibited considerably higher toxicities. Consequently, prioritizing the control of diesel vehicles and off-road diesel machinery would yield the greatest near-term health benefits across most countries. It should also be noted that international shipping was not included in this analysis, leading to an underestimation of off-road emissions, particularly in coastal regions. Given the stronger cytotoxicity of shipping-related particles [35], the RPAE contribution of transport sources, and thus the number of transport-dominated countries, may have been underestimated. As global motorization accelerates, the contribution of transport to RPAE is expected to rise. Coupled with the proximity of transport emissions to dense populations, traffic emission control therefore emerges as an increasingly decisive factor in protecting urban public health.

While our analysis highlights the shifts in dominant sources worldwide, it should be viewed as an illustration of our diagnostic framework rather than a prescriptive control blueprint. Each country needs to tailor its air pollution control strategy to its specific socioeconomic context and source composition, using toxicity-weighted metrics to identify sectors where reductions can deliver the greatest health benefits. Within this process, spatial heterogeneity is equally critical since dominant sources may differ across provinces, states, or even within individual cities. For instance, in northwestern China, residential combustion was the primary contributor to RPAE, whereas in eastern provinces such as Shanghai, industrial and transport emissions dominated [26]. These patterns are broadly similar to those in many LMICs and high-income countries, respectively [28]. Such regional contrasts underscore the need for multiscale approaches that integrate national toxicity-weighted emission inventories, regional source apportionment, and population distribution, thereby supporting the formulation of context-specific toxicity-informed emission control strategies.

## DISCUSSION

This study established the first global inventory of toxicity-adjusted PM_2.5_ emissions, offering new metrics that are potentially more closely aligned with health relevance and can enrich assessments of anthropogenic PM_2.5_ emission control strategies worldwide. Our findings reveal profound disparities in energy use, PM_2.5_ emissions, and RPAE across regions and socioeconomic contexts, with LMICs bearing the most toxic emissions, especially those in sub-Saharan Africa. Global energy use remains heavily skewed towards wealthier nations, yet RPAE disproportionately falls upon poorer populations, exposing a clear energy and environmental injustice. The discrepancy between PM_2.5_ and RPAE hotspots suggests that mass-based control strategies may obscure critical disparities in the potential health impacts of different sources, highlighting the need for toxicity-informed prioritization in air pollution control policies. Accounting for unequal toxicity leads to a realignment of priority sources worldwide: many countries shift from power- or industry-dominated to residential-dominated, whereas a few upper-middle- and high-income countries remain or shift to industry- or transport-dominated. Collectively, these results argue for developing a toxicity-informed understanding of air pollution, which could potentially provide a global framework for designing locally adapted emission control strategies that better address health impacts and reduce inequities. For example, McDuffie *et al.* [28] estimated source-specific PM_2.5_-attributable mortality at multiple spatial scales under the assumption of equal toxicity, finding that regions with large anthropogenic contributions, particularly from residential and industrial combustion sources, generally had the highest attributable deaths, broadly consistent with our results. Our findings suggest that incorporating unequal toxicities would amplify health burdens in regions dominated by high-toxicity sources while attenuating them elsewhere, implying that spatial inequities in PM_2.5_-attributable health impacts may be substantially underestimated under equitoxicity frameworks.

The world now faces a dual challenge of mitigating climate change (e.g. through carbon neutrality [36]) and reducing the health impacts of air pollution, as reflected in the WHO’s target of halving the global health burden related to air pollution by 2040. Achieving both goals will benefit from approaches that promote synergies between climate and health objectives, and incorporating toxicity into emission control policies provides a practical means to strengthen this alignment. Beyond the dominant contribution to RPAE, residential solid fuel combustion is also a major driver of severe indoor air pollution, underscoring its importance as a priority for air pollution control [37]. However, in the context of climate change mitigation, biomass is often regarded as a near-zero-carbon option [38], yet our findings show that PM_2.5_ from traditional biomass combustion exhibits the highest toxicity among major anthropogenic sources [23,26], indicating that strategies guided solely by climate criteria or health impacts of air pollution may inadvertently misalign the two goals. Biomass use, however, is highly heterogeneous: pelletized biomass burned in high-efficiency, well-ventilated stoves and boilers achieves both lower emissions and much lower particle toxicity than traditional stoves or open fires [26,39]. Such improvements in both fuel form and combustion technology offer a realistic path towards clean and sustainable energy transitions that jointly advance decarbonization and public health [36]. Nevertheless, residential biomass combustion remains the default fuel for many disadvantaged populations because of its low cost and readily accessible nature. Without transformative policy and infrastructural support, transitioning to clean fuels and modern stoves remains impractical in LMICs, risking further widening inequities in energy burden [40]. Overcoming this low-energy–high-toxicity (high RPAE) lock-in demands a sustained global momentum (e.g. China’s Clean Stove Project in developing countries announced at the United Nations [41]), anchored in international financing, technology diffusion, and equitable policy frameworks, to enable LMICs to leapfrog towards cleaner and more sustainable energy systems.

In the broader toxicological literature, PM_2.5_ toxicity has been characterized using a range of complementary approaches, including acellular oxidative potential (OP) assays and the cell-based assays adopted in this study. Acellular OP metrics characterize particle-bound reactive oxygen species (ROS) and the capacity of particles to catalytically generate ROS, and often exhibit strong assay-specific sensitivities to particular components, with dithiothreitol being more responsive to highly oxidized organics and ascorbic acid more sensitive to redox-active metals such as iron and copper [42]. In contrast, cellular assays integrate multiple biological processes and measure the biological responses of cells to particles. For example, PAHs typically show limited activity in acellular OP assays but can exert substantial toxicity following metabolic activation within cells [13]. In this study, we adopt a cell-based framework to enable systematic, source-resolved comparisons of RPs across emission sources at the global scale, while emphasizing that cellular and acellular metrics probe distinct but complementary mechanistic dimensions of PM_2.5_ toxicity and may therefore yield different component or source attributions.

Although cellular and acellular assays probe different mechanisms of PM_2.5_ toxicity, several findings from previous OP-based source apportionment studies are broadly consistent with our findings in this study. For example, OP measurements in ambient particles across Europe highlighted the high toxicity of traffic-related emissions, particularly brake wear, as well as combustion-related sources [43,44]. In particular, both lines of evidence suggest that reducing biomass combustion emissions is critical for lowering the toxic potential of ambient PM_2.5_ in Europe. Such convergence likely reflects the common ROS-related basis underlying both cellular and acellular toxicity metrics. Future work is needed to develop cross-assay-comparable toxicity metrics that integrate multiple endpoints and can be more directly linked to epidemiological evidence.

Several limitations of this study should be noted. First, the toxicological profiles used here do not yet encompass all anthropogenic sources. Future studies should further broaden the source coverage to include sectors with substantial emissions or potentially high toxic potencies, especially those strongly affected by climate change, such as wildfires [45,46] and windblown sand and dust [28,47]. Second, the global toxicity-adjusted emission inventory was established by applying toxicological profiles derived from Chinese sources. While China’s complex source characteristics broadly capture those in both developing and developed countries, region-specific variations may still exist. Expanding measurements that account for differences in fuels and technologies worldwide is needed. To evaluate the robustness of the framework, we conducted a comprehensive comparison with another global emission inventory, showing high consistency across global, regional, and national RP estimates obtained in this study (Note S1, Figs S12 and S13, and Table S4). Third, the RPs derived from A549 cellular assays reflect intrinsic toxicity rather than population-level health outcomes. Integrating toxicity-adjusted metrics with epidemiological analyses is essential for establishing quantitative links between emission toxicity and population health risks.

Despite these limitations, our dataset represents the best currently available estimate of toxicity-adjusted PM_2.5_ emissions on a global scale. Compared to the traditional assumption of equal toxicity for all PM_2.5_, this framework provides a more biologically grounded representation of the potential impacts of PM_2.5_ emissions on global health. Toxicity-informed metrics can complement existing indicators to guide policies and investments towards energy and technological transitions that enhance health benefits, equity, and sustainability. Embedding these metrics into global energy, environment, and health initiatives would strengthen coherence between air quality management and public health protection. As toxicological evidence and modelling capabilities continue to advance and as toxicity-informed metrics are validated in carefully designed epidemiologic studies, this framework could underpin a new generation of global air quality assessments that better align emission control policies with health outcomes.

## METHODS

### Toxicological profiles of PM_2.5_ emissions

The source-specific toxicological profiles of primary PM_2.5_ emissions were adopted from our previous comprehensive experimental study in China [26]. In that work, hundreds of representative emission samples were collected from major anthropogenic sources, including power plants, industry, residential combustion, and transport. The toxic potencies of PM_2.5_ were quantified using human epithelial A549 cell lines under two widely applied biological endpoints: OS and CT. The concentrations, inducing a 1.5-fold increase in ROS (EC_1.5_, effective concentration) and a 20% reduction in cell viability (IC_20_, inhibitory concentration) relative to the control, were used as quantitative indicators of OS and cytotoxic potencies, respectively. Since PM_2.5_ emitted from CFPPs exhibited the lowest toxicity among major sources, it was used as the reference to calculate the RP of each source *s* as


(1) \begin{eqnarray*}
{\mathrm{R}}{{\mathrm{P}}}_{{\mathrm{EP}},s} = \frac{{{\mathrm{Toxic\ potenc}}{{\mathrm{y}}}_{{\mathrm{EP}},{\mathrm{CFPPs}}}}}{{{\mathrm{Toxic\ potenc}}{{\mathrm{y}}}_{{\mathrm{EP}},s}}},
\end{eqnarray*}

where EP denotes the biological endpoint. Higher RP values indicate stronger toxic potency. Detailed source-specific relative RP values were adopted from our previous work [26], where the full dataset is provided in Tables S1 and S2 of that publication. A concise summary of the RP values used in this study is given in Table S3.

### Global anthropogenic emission inventory of PM_2.5_

Anthropogenic emissions of primary PM_2.5_ in 2015 were obtained from the ECLIPSE (Evaluating the Climate and Air Quality Impacts of Short-Lived Pollutants) V6b dataset (https://iiasa.ac.at/models-tools-data/global-emission-fields-of-air-pollutants-and-ghgs), developed by the Greenhouse Gas and Air Pollution Interactions and Synergies model of the International Institute for Applied Systems Analysis [27]. The dataset provides sector-, fuel-, and technology-specific emissions of 180 regions worldwide, mostly divided by country, with large countries such as China and India further resolved into provinces or states, and some small countries aggregated into broader regions (e.g. Western and Eastern Africa).

For countries without explicit national estimates in ECLIPSE, we applied a scaling approach using the sectoral proportions of PM_2.5_ emissions reported in the Emissions Database for Global Atmospheric Research (EDGAR) v8.1 inventory [48]. Specifically, for each missing country, the fractional contributions of each sector in EDGAR (national sectoral PM_2.5_/regional sectoral PM_2.5_) were multiplied by the sectoral emissions of the corresponding region from the ECLIPSE dataset to estimate national, sector-resolved emissions. The resulting dataset covers 197 countries, following the Global Burden of Disease (GBD) framework [49] (204 countries in total) after excluding territories absent from both inventories or with zero reported emissions. Following the GBD regional classification, the 21 original regions were consolidated into 14 world regions (e.g. merging the four sub-Saharan subregions) to simplify the analysis.

The ECLIPSE dataset integrates detailed activity data (e.g. energy consumption and production yields), emission factors, and end-of-pipe control measures across 304 anthropogenic subsectors. It encompasses a wide range of industrial processes such as sintering, pelletizing, blast furnaces, and electric arc furnaces in the iron and steel industry. This high level of sectoral details enables a direct and systematic linkage between emission sources and toxicological profiles, providing a robust foundation for the establishment of the global toxicity-adjusted PM_2.5_ emission inventory. It is noteworthy that open burning of biomass is included in the ECLIPSE dataset as an anthropogenic agricultural source, whereas natural wildfires are not, which requires further investigation.

### Estimation of relative potency-adjusted PM_2.5_ emissions

Country-level RPAEs were estimated by integrating the sectoral PM_2.5_ mass emissions with source-specific toxicological profiles described above. For each country *c*, the RPAEs were estimated as the sum of sectoral emissions weighted by their corresponding RPs based on the concentration addition model [50]:


(2) \begin{eqnarray*}
{\mathrm{RPA}}{{\mathrm{E}}}_{{\mathrm{EP}},c} = \mathop \sum \limits_s {\mathrm{Emi}}{{\mathrm{s}}}_{c,s} \times {\mathrm{R}}{{\mathrm{P}}}_{{\mathrm{EP}},s},
\end{eqnarray*}

where Emis*_c,s_* denotes the annual PM_2.5_ mass emissions from sector *s* in country *c*, EP represents either OS or CT.

The emission sources sampled in China covered a wide range of fuels and technologies that represent most major anthropogenic emission types worldwide. Owing to the country’s diverse energy and industrial structures, these profiles capture source characteristics in both developing and developed countries, which provides a consistent basis for the global toxicity-adjusted emissions. For industrial sources, the toxicological profile covers the majority of emission-intensive industries worldwide, particularly those characterized by high emissions of polycyclic aromatic hydrocarbons and toxic metals, such as iron and steel and non-ferrous smelting industries. Residential combustion sources are also comprehensively represented, including bituminous coal, anthracite coal, wood, and major agricultural residues such as rice, wheat, corn, and soybean straws, which together capture the dominant fuel types across regions. Nevertheless, certain region-specific sources (e.g. wildfires and unconventional fuels) are not yet included, and extending measurements to these remains important. In the absence of test data, applying existing source profiles to analogous sources is a common practice in global emission inventory development. Here, the toxic potency of open biomass burning was approximated using that of residential biomass combustion. In spite of that, the compiled profiles encompass nearly all major anthropogenic PM_2.5_ sources on a global scale and are considered broadly representative of current emission characteristics.

The spatial distribution of RPAE was developed following the method of Feng *et al.* [51]. For each country, region, or grid, the RP of PM_2.5_ emissions was estimated based on the relative contribution of sources (C*_s_*) using the following equation:


(3) \begin{eqnarray*}
{\mathrm{R}}{{\mathrm{P}}}_{{\mathrm{EP}}} = \mathop \sum \limits_s {{\mathrm{C}}}_s \times {\mathrm{R}}{{\mathrm{P}}}_{{\mathrm{EP}},s}.
\end{eqnarray*}

### Decomposition of per-capita relative potency-adjusted PM_2.5_ emissions

To examine the drivers of cross-country disparities in RPAE, national per-capita RPAE was decomposed into three multiplicative components. For each country *c*, per-capita RPAE was expressed as


(4) \begin{eqnarray*}
\frac{{{\mathrm{RPA}}{{\mathrm{E}}}_{{\mathrm{EP}},c}}}{{{\mathrm{po}}{{\mathrm{p}}}_c}} = \frac{{{{\mathrm{E}}}_c}}{{{\mathrm{po}}{{\mathrm{p}}}_c}} \times \frac{{{\mathrm{Emi}}{{\mathrm{s}}}_c}}{{{{\mathrm{E}}}_c}} \times {\mathrm{R}}{{\mathrm{P}}}_{{\mathrm{EP}},c},
\end{eqnarray*}

where E*_c_* denotes the energy consumption and pop*_c_* represents the population in country *c*. The three terms on the right-hand side correspond to per-capita energy use, emission intensity (PM_2.5_ emissions per unit of energy use), and RP of emissions.

### Inequity measure

To quantify the inequity in energy use, PM_2.5_ emissions, and toxicity-adjusted emissions across income groups, we employed the CI, a widely used measure of socioeconomic inequity [52]. The CI quantifies the degree to which a specific indicator (e.g. energy consumption) is concentrated among populations with different income levels. The CI is derived from the concentration curve, which plots the cumulative percentage of a specific indicator on the *y*-axis against the cumulative percentage of the population ranked by income on the *x*-axis. The formula for the CI is expressed as


(5) \begin{eqnarray*}
{\mathrm{CI}} = \frac{2}{\mu }\mathop \sum \limits_{i = 1}^N {w}_i{K}_i{R}_i - 1,
\end{eqnarray*}

where ${K}_i$ is the indicator value for country *i* (e.g. per-capita energy use), ${w}_i$ is the population share of country *i* worldwide, $\mu $ is the population-weighted mean of the indicator, and ${R}_i$ represents the fractional income rank of country *i*, calculated as


(6) \begin{eqnarray*}
{R}_i = \ \mathop \sum \limits_{j = 1}^{i - 1} {w}_j + \ \frac{1}{2}{w}_i,
\end{eqnarray*}

with countries ordered by GDP per capita [as purchasing power parity (PPP) in current international dollars reported by the World Bank (https://data.worldbank.org/)] from lowest to highest. CI ranges from −1 to 1, with positive values indicating a concentration among wealthier populations and negative values indicating a concentration among poorer populations. A CI value of 0 denotes the absence of inequity across income groups. Unlike the Gini coefficient, which measures overall inequality of certain indicators, CI specifically captures income-related disparities [34,53].

## Supplementary Material

nwag301_Supplemental_Files

## Data Availability

The country-level and region-level source-specific PM_2.5_ emissions, RPAE, and RP of PM_2.5_ emissions are provided in the Supplementary Data.
