# Supplementary material for: Toxicity-informed control of global PM2.5 emissions
Source: Natl Sci Rev. 2026 May 21;13(11):nwag301. doi: 10.1093/nsr/nwag301 (PMC13296561; doi:10.1093/nsr/nwag301)
Supplement: nwag301_Supplemental_Files [file nwag301_supplemental_files.zip › SI.pdf]

Supplementary Information for

“Toxicity-Informed Control of Global $PM_{2.5}$ Emissions”

This PDF file includes:

Supplementary Note 1

Supplementary Figs 1–13

Supplementary Tables 1–4

Contents

Supplementary Note 1 Comparison of this study and the results based on the EDGAR dataset

Supplementary Fig.1 Global spatial distribution of $PM_{2.5}$ emissions in 2015. The national emissions are from ECLIPSE (Evaluating the Climate and Air Quality Impacts of Short-Lived Pollutants) V6b dataset and allocated into 0.5° × 0.5° following the method developed by Feng et al.

Supplementary Fig.2 Global distributions and source contributions of toxicity-adjusted $PM_{2.5}$ emissions in 2015. a, Global distribution of $RPAE_{CT}$. **b**, Global distribution of RP_{CT} of $PM_{2.5}$ emissions. **c–d**, RP_{CT}, relative contributions of major sources to $PM_{2.5}$ mass and $RPAE_{CT}$, and global share of country- or region-specific emissions: **c**, across 14 world regions and the global average; **d**, for the top ten countries ranked by $PM_{2.5}$ mass emissions.

Supplementary Fig.3 Relationships between sectoral contributions and toxic potencies of national $PM_{2.5}$ emissions. Scatter plots show relative contributions versus RP_{OS} (top) and RP_{CT} (bottom). Panels from left to right correspond to power plants, industry, residential combustion, transport, and open burning of biomass.

Supplementary Fig.4 Global distribution of per-capita national $PM_{2.5}$ emissions and RP_{OS}. Countries were grouped into low, medium, and high categories using the 25th and 75th percentiles of per-capita emissions, and thresholds of 10 and 25 for RP_{OS}. The color scale shows each country's combined category, and the percentages in the legend indicate the proportion of countries in each category.

Supplementary Fig.5 Regional relationships between income level and $PM_{2.5}$ toxicity. Relationships between GDP per capita (as purchasing power parity (PPP), current international dollars, obtained from the World Bank (https://data.worldbank.org/)) and relative potency for oxidative stress (RP_{OS}) of $PM_{2.5}$ emissions across major world regions in 2015. Bubble size denotes $RPAE_{OS}$, and colour denotes per-capita emissions. Dashed lines show global average GDP per capita and RP_{OS}.

Supplementary Fig.6 Income-group disparities in residential energy consumption per capita (GJ). Data are plotted as box-and-whiskers (The line of the box is the median, the edges of the boxes are the 25th and 75th percentiles, and the lengths of the whiskers are within 1.5 times the interquartile range). Each box plot represents the distribution within the income category. Pie charts show the relative shares of coal, oil and natural gas, and biomass by income category.

Supplementary Fig.7 Income-group disparities in energy structure, emission control, emission intensity, and $PM_{2.5}$ emissions per capita. a, Residential energy share (%). Data are plotted as box-and-whiskers (The line of the box is the median, the edges of the boxes are the 25th and 75th percentiles, and the lengths of the whiskers are within 1.5 times the interquartile range). **b**, Integrated removal rate of $PM_{2.5}$ emissions by control measures. **c**, $PM_{2.5}$ emission per unit energy use (kg/GJ). **d**, $PM_{2.5}$ emissions per capita (kg).

Supplementary Fig.8 Income-group disparities in relative potencies and $RPAE_{CT}$ per capita. a, RP_{OS} of $PM_{2.5}$ emissions. Data are plotted as box-and-whiskers (The line of the box is the median, the edges of the boxes are the 25th and 75th percentiles, and the lengths of the whiskers are within 1.5 times

the interquartile range). **b**, RP_{CT} of $PM_{2.5}$ emissions. **c**, $RPAE_{CT}$ per capita (kg).

Supplementary Fig.9 Relationship between residential energy share and relative potency for oxidative stress (RP_{OS}) of $PM_{2.5}$ emissions across income groups. Each point represents a country, coloured by income group (low, lower middle, upper middle, and high). The solid red curve and shaded area denote the fitted nonlinear regression and its 95% confidence interval, respectively. Kernel density estimates along the top and right margins show the distributions of residential energy shares and RP_{OS} values, respectively, for each income group.

Supplementary Fig.10 Global concentration curves of income, energy use, $PM_{2.5}$ emissions, and toxicity-adjusted emissions. Concentration curves depict cumulative population shares (ranked by income per capita) against cumulative shares of variables.

Supplementary Fig.11 Income-group disparities in dominant sources. Bars indicate the proportion of countries where each source category is dominant in national $PM_{2.5}$ mass emissions and toxicity-adjusted emissions. The numbers on the bars denote the corresponding counts of countries.

Supplementary Fig.12 Comparison of global and regional relative potencies of $PM_{2.5}$ emissions between estimations based on ECLIPSE (this study) and EDGAR datasets: a, RP_{OS}; b, RP_{CT}. The data represent arithmetic means, and the error bars indicate ±1 standard deviation (SD).

Supplementary Fig.13 Comparison of national relative potencies between estimations based on ECLIPSE (this study) and EDGAR datasets. Scatter plots show the correspondence of RP_{OS} (a) and RP_{CT} (b) between the two inventories. The dashed line denotes the 1:1 reference, and the shaded empirical band around it (90% coverage) highlights the distribution of residuals. The top ten $PM_{2.5}$-emitting countries (e.g., China, India, and the United States) are highlighted in red, while the other labeled countries.

Supplementary Table 1 Member countries and territories of 14 world regions. The regional grouping follows the Global Burden of Disease (GBD) framework, but with several neighboring regions merged to simplify the analysis.

Supplementary Table 2 World countries by income group (World Bank classification). Countries marked with an asterisk (*) are not assigned an official income category by the World Bank; their income groups were estimated based on additional publicly available information.

Supplementary Table 3 Relative potency (dimensionless) of major anthropogenic sources.

Supplementary Table 4 Comparison of $PM_{2.5}$ emissions, $RPAE_{OS}$, $RPAE_{CT}$, RP_{OS}, and RP_{CT} between estimations based on ECLIPSE (this study) and EDGAR datasets.

Supplementary Notes

1. Comparison of this study and the results based on the EDGAR dataset

To evaluate the robustness and transferability of our toxicity-informed framework, we compared the toxicity-adjusted emissions derived from the ECLIPSE v6b dataset with those based on the Emission Database for Global Atmospheric Research (EDGAR v8.1) [1]. Here, we estimated the toxicity-adjusted emissions derived from the EDGAR inventory by integrating the same source-specific toxicological profiles as those used in this study (see **Methods**). By matching 29 emission sectors with corresponding toxicological profiles, we obtained RPAE and RP values of $PM_{2.5}$ emissions based on the EDGAR dataset. Considering the differences in total $PM_{2.5}$ emissions in different datasets owing to variations in activity data, emission factors, and end-of-pipe control measures, our major objective is not to compare the absolute RPAE, but rather to examine the differences in the estimated RP values, which play a critical role in the proposed toxicity-informed framework.

In terms of global toxicity-adjusted $PM_{2.5}$ emissions, despite certain differences in total amounts, which are largely attributable to the differences in total $PM_{2.5}$ mass emissions, both datasets produced highly consistent global patterns of RP_{OS} and RP_{CT}. The absolute deviations of RP_{OS} and RP_{CT} between the two datasets were 0.4 and -1.8, corresponding to relative differences of 2.8% and –7.1%, respectively (**Supplementary Table 4**). These small discrepancies indicate that our toxicity-adjusted approach is robust to the choice of emission inventory.

At the regional scale, results derived from the two datasets exhibited strong spatial consistency, especially in identifying toxicity hotspots in Sub-Saharan Africa and other regions dominated by traditional biomass combustion (**Supplementary Fig.12**). Minor discrepancies in some regions stem from differences in emission factors and sectoral classifications used between the two datasets. Nevertheless, the overall spatial and cross-regional patterns remained stable, demonstrating that the toxicity-informed framework captures the essential distribution of $PM_{2.5}$ toxicity regardless of the selection of emission datasets.

At the national scale, the results derived from the two inventories showed overall good agreement, indicating that the toxicity-informed framework performs consistently across datasets. For most countries, national estimates of RP_{OS} and RP_{CT} were broadly comparable, with normalized mean bias of 0.1% and 13.0%, respectively. A few countries exhibited larger deviations between the two datasets, which primarily stem from variations in sectoral compositions. Even so, the overall discrepancies remain within acceptable ranges. The correlation coefficients (R^2 = 0.85 for RP_{OS} and 0.76 for RP_{CT}) confirm a high level of consistency. For major emitting countries such as China, India, and the United States, the agreement between inventories was particularly strong, with errors usually smaller than the global average (**Supplementary Fig.13**). In the case of the United States, the slightly higher industrial emissions in EDGAR and higher residential emissions in ECLIPSE led to minor differences in estimated RP values, but the overall cross-country patterns and RP gradients were largely consistent between the two inventories.

In summary, although global emission inventories differ in methodology and data source, the toxicity-informed framework presented here demonstrates strong transferability and robustness. Its ability to produce consistent global and regional toxicity patterns across independent datasets validates its applicability for multi-scale assessments, e.g., from global to national levels, and highlights its potential to

inform health- and sustainability-oriented energy transition and emission control strategies.

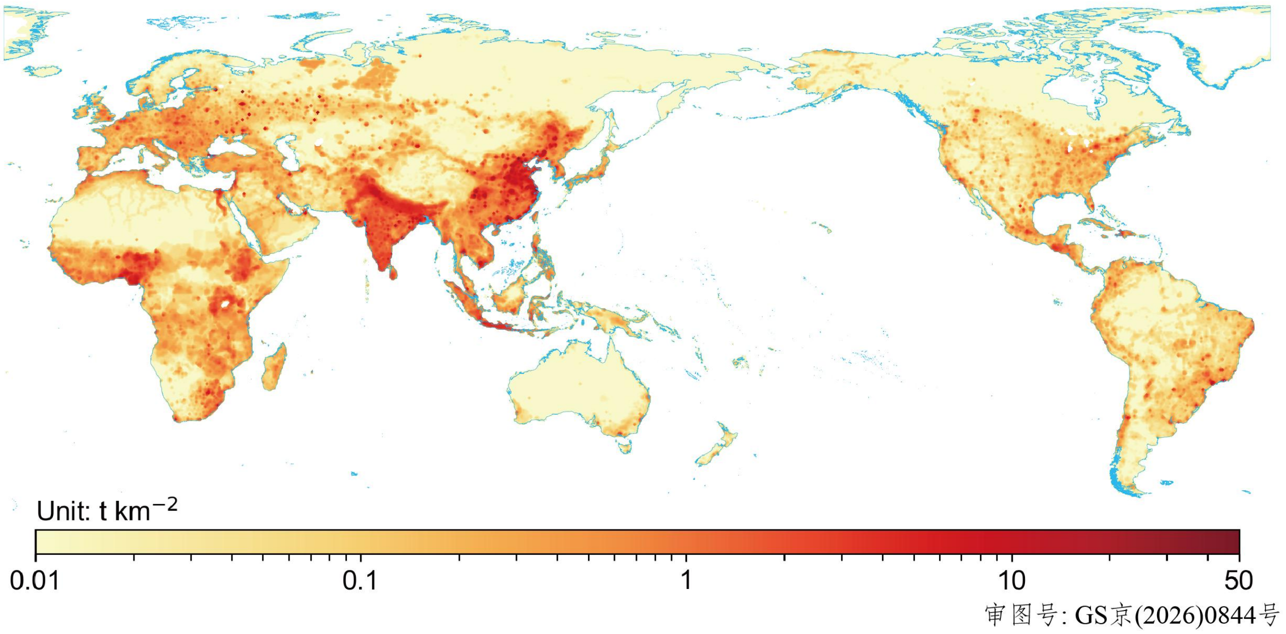

Supplementary Fig.1 Global spatial distribution of $PM_{2.5}$ emissions in 2015. The national emissions are from ECLIPSE (Evaluating the Climate and Air Quality Impacts of Short-Lived Pollutants) V6b dataset [2] and allocated into 0.5° × 0.5° following the method developed by Feng et al. [3].

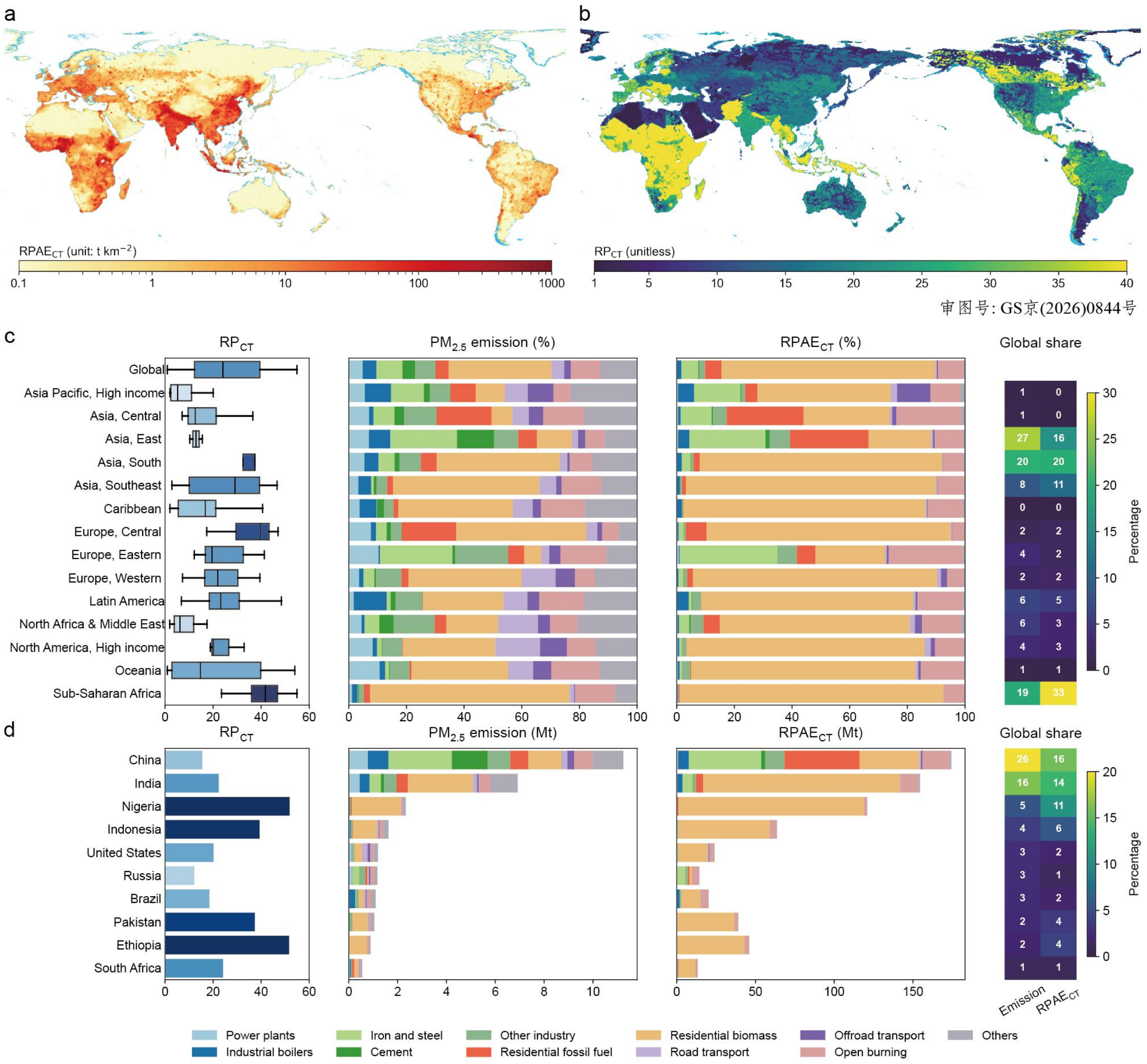

Supplementary Fig.2 Global distributions and source contributions of toxicity-adjusted $PM_{2.5}$ emissions in 2015. a, Global distribution of $RPAE_{CT}$. **b**, Global distribution of RP_{CT} of $PM_{2.5}$ emissions. **c–d**, RP_{CT}, relative contributions of major sources to $PM_{2.5}$ mass and $RPAE_{CT}$, and global share of country- or region-specific emissions: **c**, across 14 world regions and the global average; **d**, for the top ten countries ranked by $PM_{2.5}$ mass emissions.

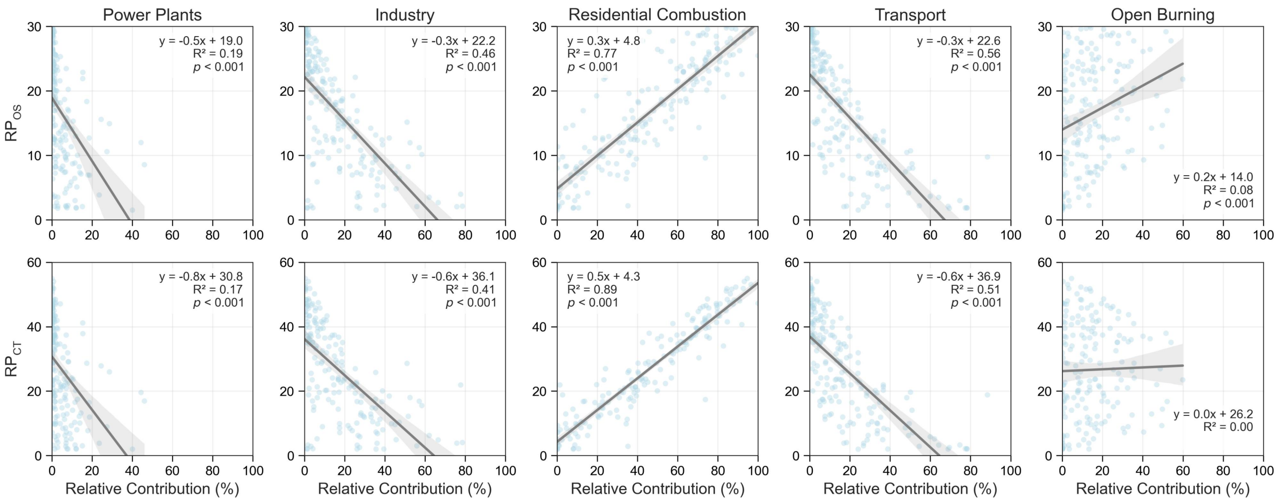

Supplementary Fig.3 Relationships between sectoral contributions and toxic potencies of national $PM_{2.5}$ emissions. Scatter plots show relative contributions versus RP_{OS} (top) and RP_{CT} (bottom). Panels from left to right correspond to power plants, industry, residential combustion, transport, and open burning of biomass.

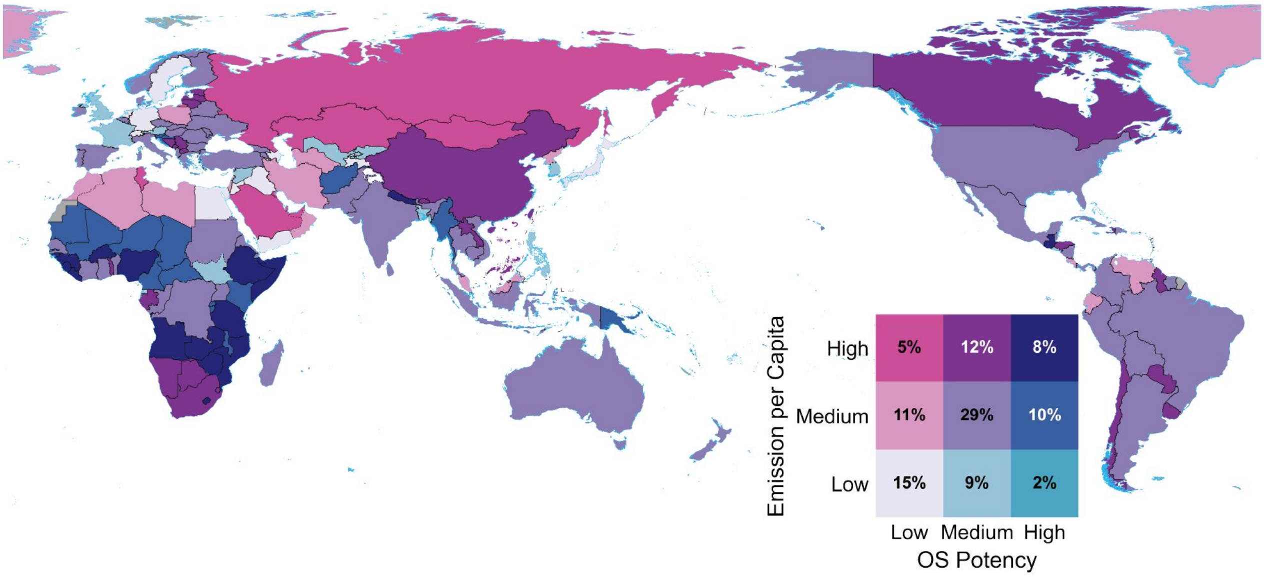

审图号: GS京(2026)0844号

Supplementary Fig.4 Global distribution of per-capita national $PM_{2.5}$ emissions and RP_{OS}. Countries were grouped into low, medium, and high categories using the 25th and 75th percentiles of per-capita emissions, and thresholds of 10 and 25 for RP_{OS}. The color scale shows each country's combined category, and the percentages in the legend indicate the proportion of countries in each category.

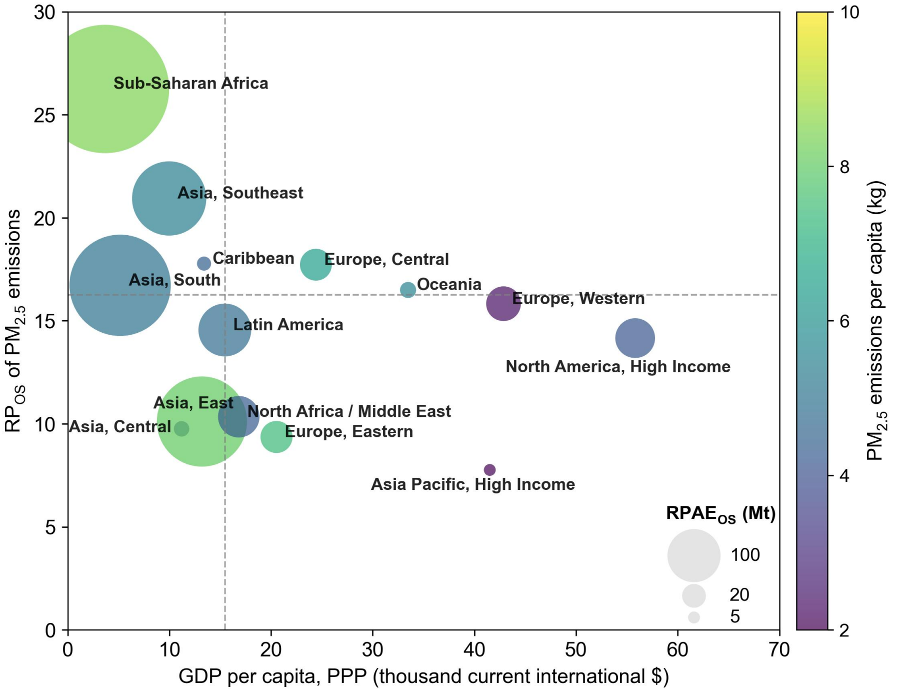

Supplementary Fig.5 Regional relationships between income level and $PM_{2.5}$ toxicity. Relationships between GDP per capita (as purchasing power parity (PPP), current international dollars, obtained from the World Bank (https://data.worldbank.org/)) and relative potency for oxidative stress (RP_{OS}) of $PM_{2.5}$ emissions across major world regions in 2015. Bubble size denotes $RPAE_{OS}$, and colour denotes per-capita emissions. Dashed lines show global average GDP per capita and RP_{OS}.

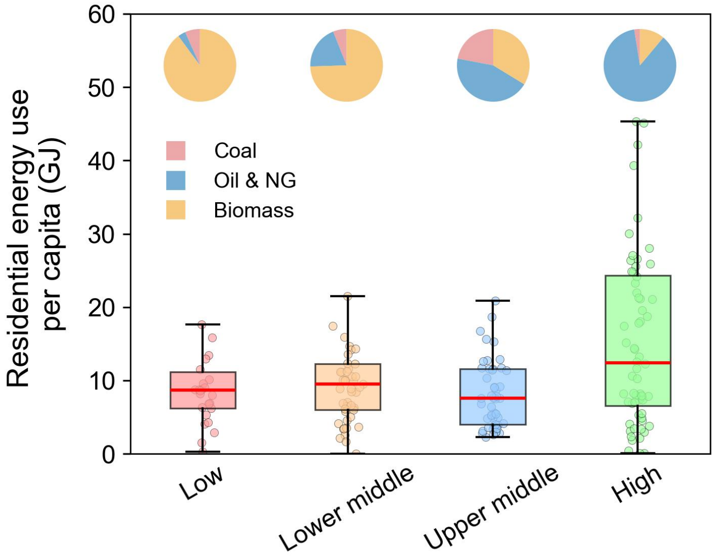

Supplementary Fig.6 Income-group disparities in residential energy consumption per capita (GJ). Data are plotted as box-and-whiskers (The line of the box is the median, the edges of the boxes are the 25th and 75th percentiles, and the lengths of the whiskers are within 1.5 times the interquartile range). Each box plot represents the distribution within the income category. Pie charts show the relative shares of coal, oil and natural gas, and biomass by income category.

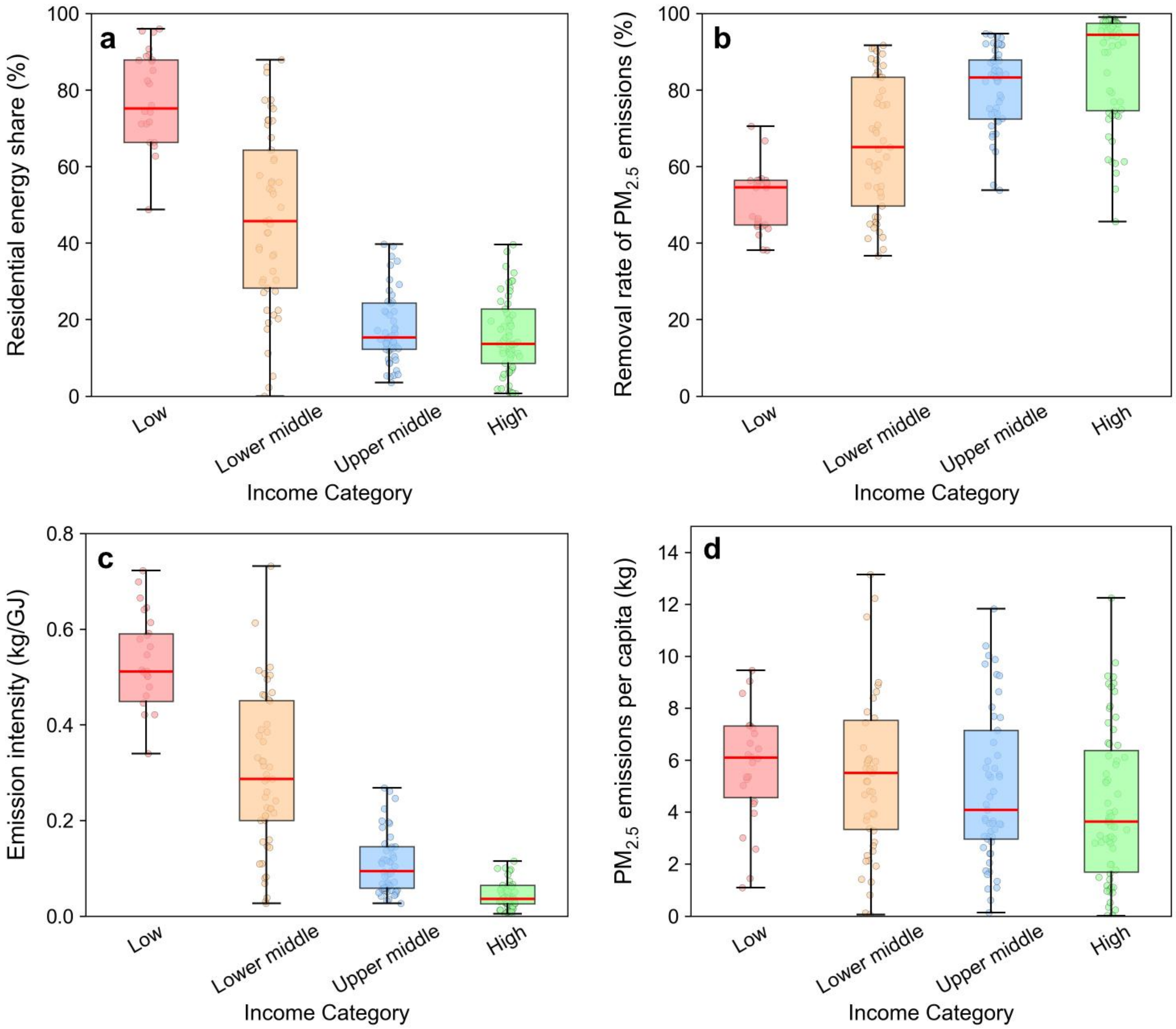

Supplementary Fig.7 Income-group disparities in energy structure, emission control, emission intensity, and $PM_{2.5}$ emissions per capita. a, Residential energy share (%). Data are plotted as box-and-whiskers (The line of the box is the median, the edges of the boxes are the 25th and 75th percentiles, and the lengths of the whiskers are within 1.5 times the interquartile range). **b**, Integrated removal rate of $PM_{2.5}$ emissions by control measures. **c**, $PM_{2.5}$ emission per unit energy use (kg/GJ). **d**, $PM_{2.5}$ emissions per capita (kg).

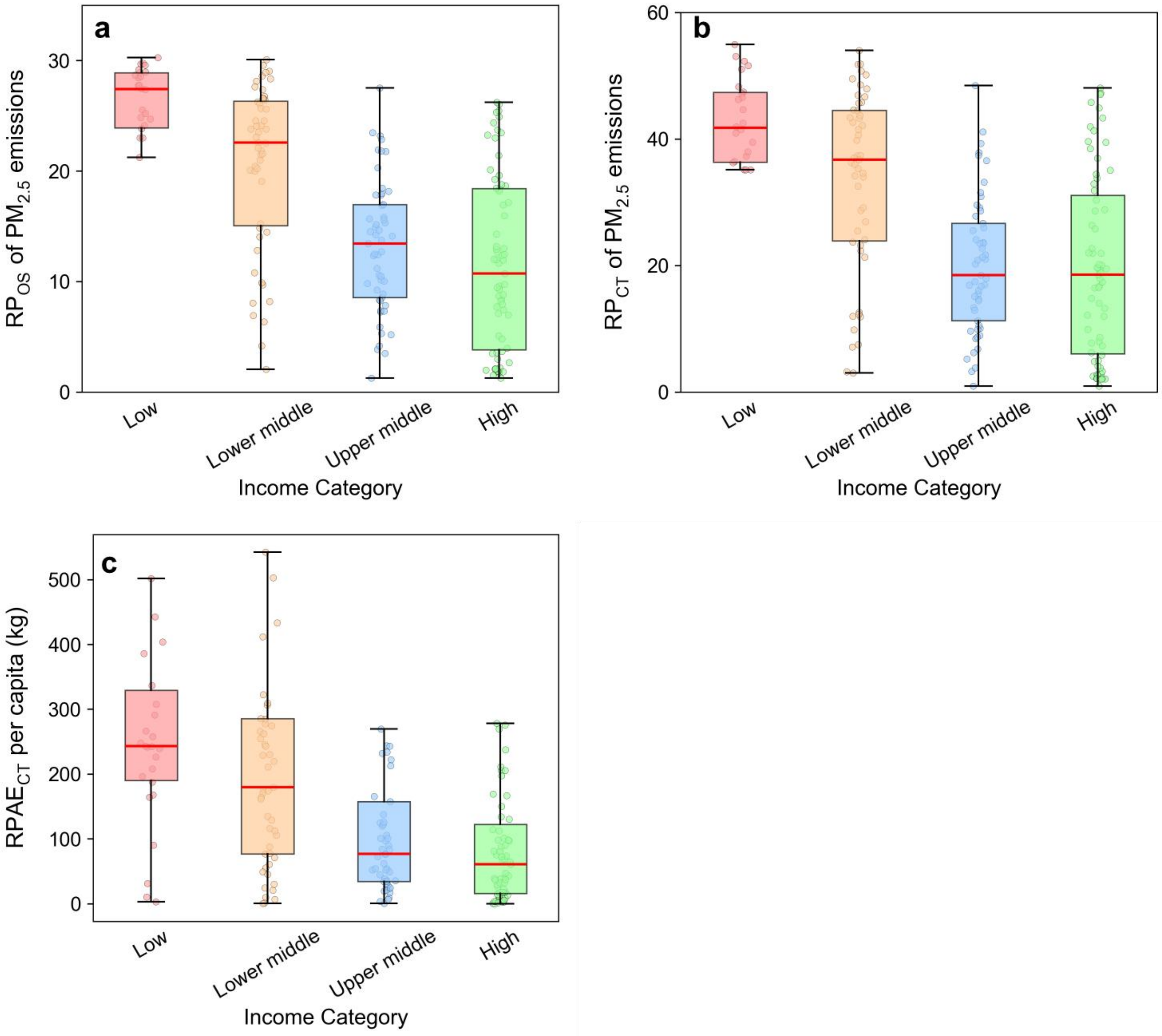

Supplementary Fig.8 Income-group disparities in relative potencies and RPAE$_{CT}$ per capita. a, RP$_{OS}$ of PM$_{2.5}$ emissions. Data are plotted as box-and-whiskers (The line of the box is the median, the edges of the boxes are the 25th and 75th percentiles, and the lengths of the whiskers are within 1.5 times the interquartile range). **b**, RP$_{CT}$ of PM$_{2.5}$ emissions. **c**, RPAE$_{CT}$ per capita (kg).

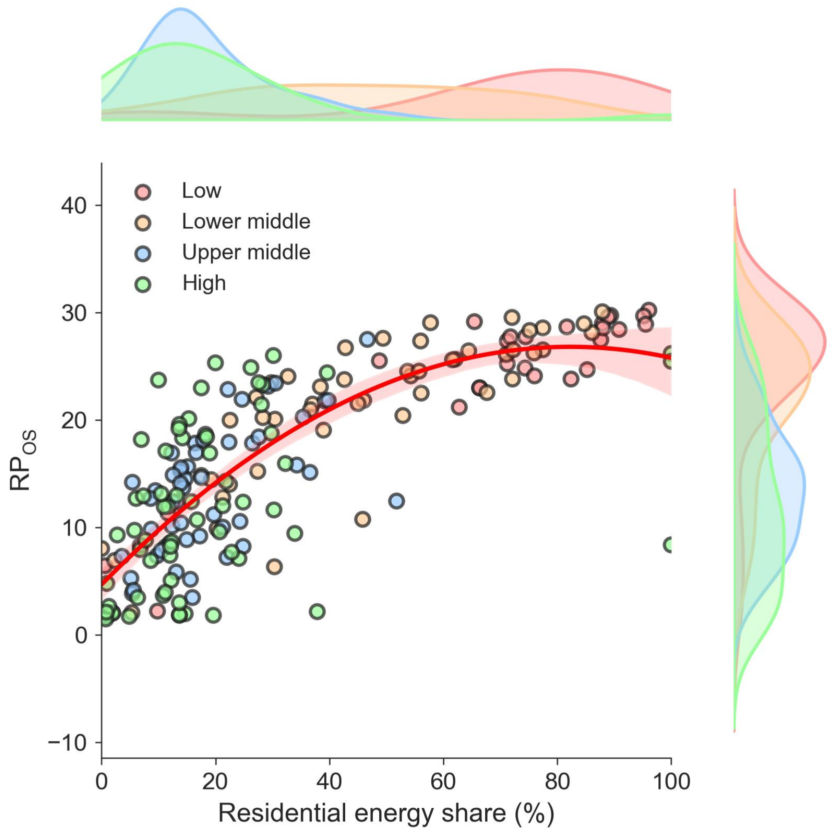

Supplementary Fig.9 Relationship between residential energy share and relative potency for oxidative stress (RP_{OS}) of $PM_{2.5}$ emissions across income groups. Each point represents a country, coloured by income group (low, lower middle, upper middle, and high). The solid red curve and shaded area denote the fitted nonlinear regression and its 95% confidence interval, respectively. Kernel density estimates along the top and right margins show the distributions of residential energy shares and RP_{OS} values, respectively, for each income group.

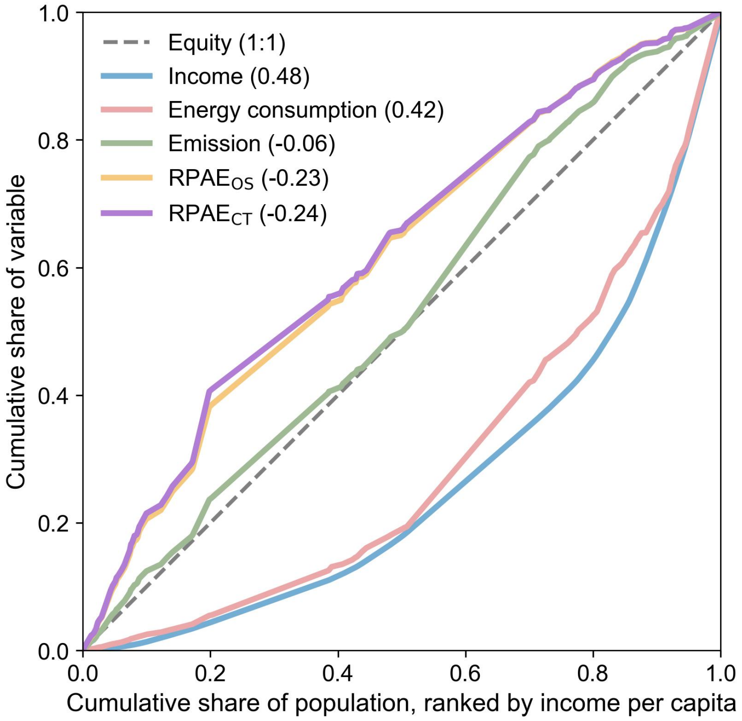

Supplementary Fig.10 Global concentration curves of income, energy use, $PM_{2.5}$ emissions, and toxicity-adjusted emissions. Concentration curves depict cumulative population shares (ranked by income per capita) against cumulative shares of variables.

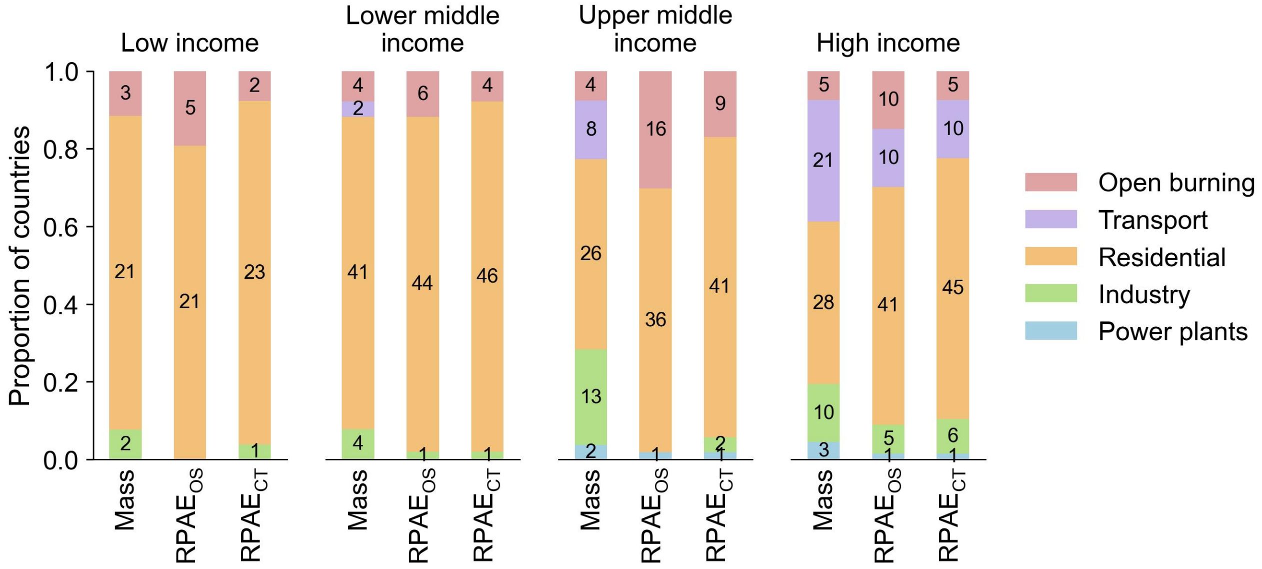

Supplementary Fig.11 Income-group disparities in dominant sources. Bars indicate the proportion of countries where each source category is dominant in national $PM_{2.5}$ mass emissions and toxicity-adjusted emissions. The numbers on the bars denote the corresponding counts of countries.

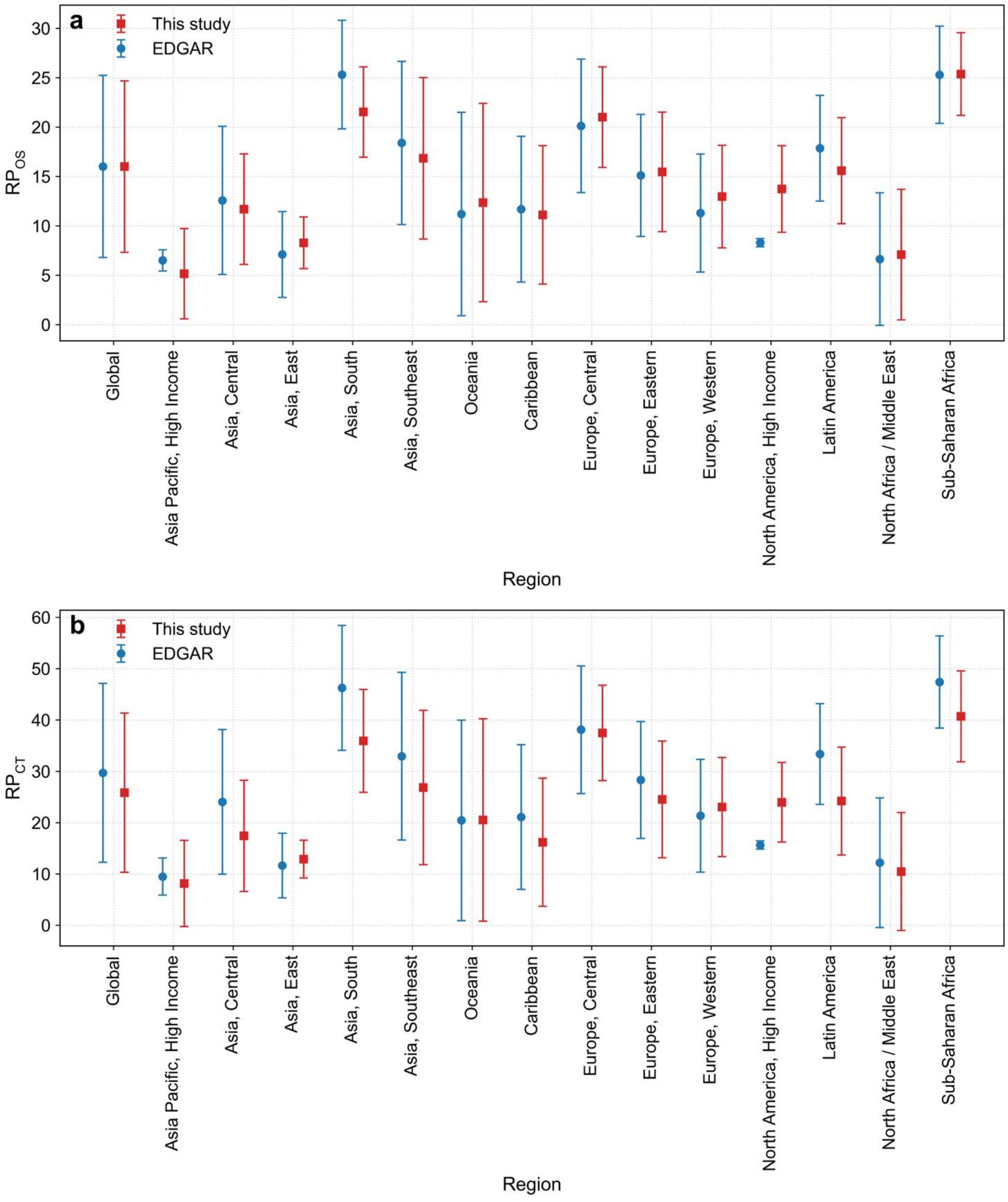

Supplementary Fig.12 Comparison of global and regional relative potencies of $PM_{2.5}$ emissions between estimations based on ECLIPSE (this study) and EDGAR datasets: a, RP_{OS}; b, RP_{CT}. The data represent arithmetic means, and the error bars indicate ±1 standard deviation (SD).

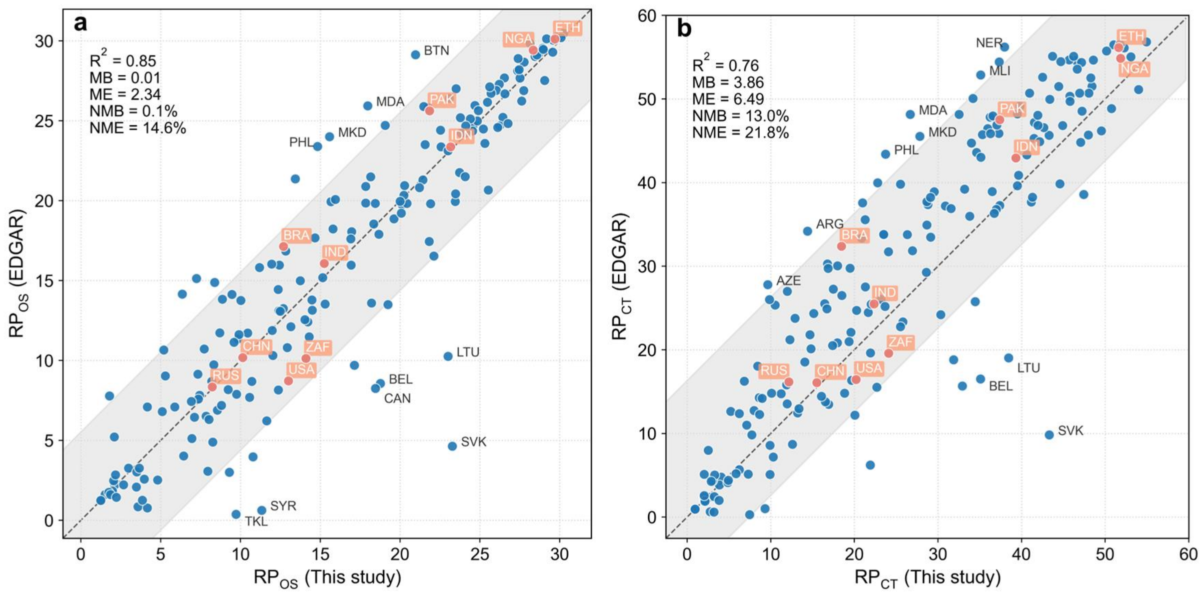

Supplementary Fig.13 Comparison of national relative potencies between estimations based on ECLIPSE (this study) and EDGAR datasets. Scatter plots show the correspondence of RP_{OS} (a) and RP_{CT} (b) between the two inventories. The dashed line denotes the 1:1 reference, and the shaded empirical band around it (90% coverage) highlights the distribution of residuals. The top ten $PM_{2.5}$-emitting countries (e.g., China, India, and the United States) are highlighted in red, while the other labeled countries.

Supplementary Table 1 Member countries and territories of 14 world regions. The regional grouping follows the Global Burden of Disease (GBD) framework, but with several neighboring regions merged to simplify the analysis.

GBD Region	Member Countries			
**Asia Pacific, High Income**	Brunei Darussalam	Japan	South Korea	Singapore
**Asia, Central**	Armenia	Azerbaijan	Georgia	Kazakhstan
	Kyrgyzstan	Mongolia	Tajikistan	Turkmenistan
	Uzbekistan			
**Asia, East**	China	North Korea		
**Asia, South**	Bangladesh	Bhutan	India	Nepal
	Pakistan			
**Asia, Southeast**	Cambodia	Indonesia	Malaysia	Mauritius
	Myanmar	Philippines	Sri Lanka	Seychelles
	Thailand	Vietnam	Laos	Maldives
	Timor-Leste			
**Oceania**	Australia	New Zealand	American Samoa	Cook Islands
	Fiji	Guam	Kiribati	Nauru
	Niue	Palau	Papua New Guinea	Samoa
	Solomon Islands	Tokelau	Tonga	Tuvalu
	Vanuatu	Federated States of Micronesia		
**Caribbean**	The Bahamas	Barbados	Belize	Bermuda
	Cuba	Dominica	Dominican Republic	Grenada
	Guyana	Haiti	Jamaica	Puerto Rico
	Saint Lucia	Suriname	US Virgin Islands	Saint Kitts and Nevis
	Trinidad and Tobago	Antigua and Barbuda	Saint Vincent and the Grenadines	
**Europe, Central**	Albania	Bulgaria	Croatia	Czech Republic
	Hungary	North Macedonia	Montenegro	Poland
	Romania	Serbia	Slovakia	Slovenia
	Bosnia and Herzegovina			
**Europe, Eastern**	Belarus	Estonia	Latvia	Lithuania
	Republic of Moldova	Russian Federation	Ukraine	
**Europe, Western**	Austria	Belgium	Cyprus	Denmark
	Finland	France	Germany	Greece
	Iceland	Ireland	Israel	Italy
	Luxembourg	Malta	Netherlands	Norway
	Portugal	Spain	Sweden	Switzerland
	United Kingdom			
**North America, High Income**	Canada	United States	Greenland	
**Latin America**	Bolivia	Ecuador	Peru	Colombia
	Costa Rica	El Salvador	Guatemala	Honduras
	Mexico	Nicaragua	Panama	Venezuela
	Argentina	Chile	Uruguay	Brazil
	Paraguay			

**North Africa / Middle East**	Afghanistan	Algeria	Bahrain	Egypt
	Iraq	Jordan	Kuwait	Lebanon
	Libya	Morocco	Oman	Qatar
	Saudi Arabia	Sudan	Syrian Arab Republic	Tunisia
	Turkey	Yemen	United Arab Emirates	Iran
**Sub-Saharan Africa**	Angola	Congo	Equatorial Guinea	Gabon
	Central African Republic		Democratic Republic of the Congo	
	Burundi	Comoros	Djibouti	Eritrea
	Ethiopia	Kenya	Madagascar	Malawi
	Mozambique	Rwanda	Somalia	South Sudan
	Uganda	Zambia	United Republic of Tanzania	
	Botswana	Lesotho	Namibia	South Africa
	Eswatini	Zimbabwe	Benin	Burkina Faso
	Cameroon	Cape Verde	Chad	Cote d'Ivoire
	The Gambia	Ghana	Guinea	Guinea-Bissau
	Liberia	Mali	Mauritania	Niger
	Nigeria	Senegal	Sierra Leone	Togo
	Sao Tome and Principe			

Supplementary Table 2 World countries by income group (World Bank classification). Countries marked with an asterisk (*) are not assigned an official income category by the World Bank; their income groups were estimated based on additional publicly available information.

Income Group	Member Countries			
**High income**	United Arab Emirates	American Samoa*	Antigua and Barbuda	Australia
	Austria	Belgium	Bulgaria	Bahrain
	The Bahamas	Bermuda	Barbados	Brunei Darussalam
	Canada	Switzerland	Chile	Cook Islands*
	Cyprus	Czech Republic	Germany	Denmark
	Spain	Estonia	Finland	France
	United Kingdom	Greece	Greenland	Guam*
	Guyana	Croatia	Hungary	Ireland
	Iceland	Israel	Italy	Japan
	Saint Kitts and Nevis	South Korea	Kuwait	Lithuania
	Luxembourg	Latvia	Malta	Niue*
	Netherlands	Norway	Nauru*	New Zealand
	Oman	Panama	Palau*	Poland
	Puerto Rico	Portugal	Qatar	Romania
	Russian Federation	Saudi Arabia	Singapore	Slovakia
	Slovenia	Sweden	Seychelles	Trinidad and Tobago
	Uruguay	United States	US Virgin Islands	
**Upper-middle income**	Albania	Argentina	Armenia	Azerbaijan
	Bosnia and Herzegovina		Belarus	Belize
	Brazil	Botswana	China	Colombia
	Costa Rica	Cuba	Dominica	Dominican Republic
	Algeria	Ecuador	Fiji*	Gabon
	Georgia	Equatorial Guinea	Grenada	Guatemala
	Indonesia	Iran	Iraq	Jamaica
	Kazakhstan	Libya	Saint Lucia	Republic of Moldova
	Maldives*	Mexico	North Macedonia	Montenegro
	Mongolia	Mauritius	Malaysia	Namibia
	Peru	Paraguay	El Salvador	Serbia
	Suriname	Thailand	Turkmenistan	Tonga*
	Turkey	Tuvalu	Ukraine	Venezuela*
	South Africa	Saint Vincent and the Grenadines		
**Lower-middle income**	Angola	Benin	Bangladesh	Bolivia
	Bhutan	Cote d'Ivoire	Cameroon	Congo
	Comoros	Cape Verde	Djibouti	Egypt
	Federated States of Micronesia*		Ghana	Guinea
	Honduras	Haiti	India	Jordan
	Kenya	Kyrgyzstan	Cambodia	Kiribati*
	Laos	Lebanon	Sri Lanka	Lesotho

	Morocco	Myanmar	Mauritania	Nigeria
	Nicaragua	Nepal	Pakistan	Philippines
	Papua New Guinea	Senegal	Solomon Islands*	Eswatini
	Sao Tome and Principe	Tajikistan	Tokelau*	Timor-Leste*
	United Republic of Tanzania		Samoa*	Tunisia
	Uzbekistan	Vietnam	Vanuatu*	Zambia
	Zimbabwe			
**Low income**	Afghanistan	Burundi	Burkina Faso	Eritrea
	Central African Republic		Democratic Republic of the Congo	
	Ethiopia	The Gambia	Guinea-Bissau	Liberia
	Madagascar	Mali	Mozambique	Malawi
	Niger	North Korea	Rwanda	Sudan
	Sierra Leone	Somalia	South Sudan	Syrian Arab Republic
	Chad	Togo	Uganda	Yemen

Supplementary Table 3 Relative potency (dimensionless) of major anthropogenic sources.

Source	RP_{OS}	RP_{CT}
Power plants	1.0–1.2	1.0–1.4
Industry	0.9–8.1	0.6–28.3
Transport	1.1–16.0	1.4–31.2
Residential	2.9–42.9	4.3–78.6

Supplementary Table 4 Comparison of $PM_{2.5}$ emissions, $RPAE_{OS}$, $RPAE_{CT}$, RP_{OS}, and RP_{CT} between estimations based on ECLIPSE (this study) and EDGAR datasets.

	$PM_{2.5}$ Emissions	$RPAE_{OS}$	$RPAE_{CT}$	RP_{OS}	RP_{CT}
ECLIPSE	42.6 Mt	691.0 Mt	1107.0 Mt	16.2	26.0
EDGAR	36.0 Mt	566.7 Mt	1000.0 Mt	15.8	27.8
Absolute difference	6.7 Mt	124.2 Mt	107.1 Mt	0.4	-1.8
Relative difference	15.6%	18.0%	9.7%	2.8%	-7.1%

References

1. Crippa M, Guizzardi D, Pagani F *et al.* Insights into the spatial distribution of global, national, and subnational greenhouse gas emissions in the Emissions Database for Global Atmospheric Research (EDGAR v8.0). *ESSD* 2024;**16**(6):2811–30. https://doi.org/10.5194/essd-16-2811-2024.

2. Klimont Z, Kupiainen K, Heyes C *et al.* Global anthropogenic emissions of particulate matter including black carbon. *Atmos Chem Phys* 2017;**17**(14):8681–723. https://doi.org/10.5194/acp-17-8681-2017.

3. Feng L, Smith SJ, Braun C *et al.* The generation of gridded emissions data for CMIP6. *Geosci Model Dev* 2020;**13**(2):461–82. https://doi.org/10.5194/gmd-13-461-2020.
